# TNF induces catabolism in human cartilaginous endplate cells in 3D agarose culture under dynamic compression

**DOI:** 10.1038/s41598-025-00538-w

**Published:** 2025-05-06

**Authors:** Katherine B. Crump, Exarchos Kanelis, Maria Segarra-Queralt, Andreu Pascuet-Fontanet, Paola Bermudez-Lekerika, Ahmad Alminnawi, Liesbet Geris, Leonidas G. Alexopoulos, Jérôme Noailly, Benjamin Gantenbein

**Affiliations:** 1 Tissue Engineering for Orthopaedics & Mechanobiology, Bone & Joint Program, Department for BioMedical Research (DBMR), Faculty of Medicine, University of Bern, 3008 Bern, Switzerland; 2 Graduate School for Cellular and Biomedical Sciences (GCB), University of Bern, 3012 Bern, Switzerland; 3 School of Mechanical Engineering, National Technical University of Athens, 15772 Zografou, Greece; 4 Protavio Ltd, 15341 Agia Paraskevi, Greece; 5 BCN Medtech, Universitat Pompeu Fabra, 08018 Barcelona, Spain; 6 GIGA In Silico Medicine, University of Liège, Liège, 4000 Belgium; 7 Skeletal Biology and Engineering Research Center, KU Leuven, 3000 Leuven, Belgium; 8 Department of Orthopaedic Surgery and Traumatology, Inselspital, Bern University Hospital, Faculty of Medicine, University of Bern, 3010 Bern, Switzerland

**Keywords:** Intervertebral disc, Cartilaginous endplate 3D culture, Agarose, Dynamic compression, Catabolism, Mechanobiology, Pro-inflammatory cytokines, Hydrogel, Biomedical engineering, Skeleton

## Abstract

Intervertebral disc (IVD) degeneration is the leading cause of low back pain in young adults, and the cartilaginous endplate (CEP) is likely to play a key role in early IVD degeneration. To elucidate the effects of pro-inflammatory cytokines on the mechanobiology of the CEP, human CEP cells were seeded into 2% agarose, dynamically compressed up to 7%, and stimulated with tumor necrosis factor (TNF). It was hypothesized that dynamic compression would be sufficient to induce anabolism, while stimulation with TNF would induce catabolism. TNF was sufficient to induce a catabolic, time-dependent response in human CEP cells through downregulation of anabolic gene expression and increased secretion of pro-inflammatory proteins associated with herniated discs, bacteria inhibition, and pain. However, 7% strain or scaffold material, agarose, may not lead to full activation of integrins and downregulation of pro-inflammatory pathways, demonstrated in part through the unchanged gene expression of integrin subunits α_5_ and β_1_.

**Supplementary Information:**

The online version contains supplementary material available at 10.1038/s41598-025-00538-w.

## Introduction

Intervertebral disc (IVD) degeneration is the main cause of low back pain (LBP) cases in young adults^1^ However, initiating risk factors are poorly understood as it is a highly multifactorial disease. The cartilaginous endplates (CEP) cover the cranial and caudal surfaces of the IVD and act to transmit compressive loads and transport water, nutrients, and waste in and out of the IVD^2^ However, with aging and degeneration, the CEP experiences structural changes and altered permeability^3^ Further, early CEP degeneration is believed to be a cause of nucleus dehydration and to play a key role in IVD degeneration, LBP, and Modic Changes (MC)^2,4,5^.

The CEP is a hyaline-like cartilage primarily composed of type II collagen (COL2A1) and proteoglycans^6^ It acts as a physical filter preventing macromolecules from entering the disc through the subchondral bone and is essential in maintaining hydration of the disc under compressive loading^5,7,8^ Additionally, the CEP allows transport of metabolites, small molecules, and waste into and out of the IVD. CEP cells are rounded, similar to the mid or superficial zone of chondrocytes found in articular cartilage (AC) and are surrounded by a randomly arranged pericellular matrix (PCM) made up of hyaluronan, proteoglycans, and type VI collagen (COL6A1)^3,9^ The CEP has a similar cell density (1.5 × 10^7^ cells/mL) to that of AC, which is much greater than that of the nucleus pulposus (NP) (4 × 10^6^ cells/mL) or annulus fibrosus (AF) (9 × 10^6^ cells, mL)^10^ Moreover, the CEP has a similar proteoglycan content to AC between 4 and 18% of dry weight^11,12^ but a lower water content of 22.1–62.4%^3,11^ compared to the 70.7% seen in AC^13^ As the IVD is considered as immune-privileged, inflammation can occur only when there is damage to the CEP or AF, such as rupture or fissure formation, allowing immune cell infiltration^14^ However, disc cells have been shown to produce pro-inflammatory cytokines, including interleukin (IL)-1, IL-6, IL-8 and IL-17, and tumor necrosis factor (TNF) even in the absence of an immune response, activating intracellular signaling pathways that drive degeneration and upregulate matrix degrading enzymes as well as neurotrophic and angiogenic factors that stimulate blood vessel and nerve fiber infiltration^15,16^.

The effects of a pro-inflammatory or catabolic microenvironment have been widely studied in NP and AF tissues, however, its importance in the CEP has only recently been recognized^2^ Mengis et al. (2024) investigated toll-like receptors (TLR) in degenerative CEP cells, finding that stimulation with TNF and IL-1β increased TLR2 expression alongside upregulated matrix metalloproteinases MMP-1, -3, -9, and − 13 as well as key inflammatory genes IL-1, IL-6, and CCL2^17^ Additionally, expression of pro-inflammatory cytokines has been demonstrated as higher in degenerative discs with adjacent MC^4,18^ Research has shown that degenerative NP and AF cells respond different to mechanical stimuli than those from a healthy disc, suggesting that pro-inflammatory cytokines alter crucial mechanotransduction pathways^19–22^ In NP cells, actin contractility has been shown to regulate nuclear-factor kappa-B (NF-κB) and downstream extracellular matrix (ECM) degradation, conveying that mechanotransduction and inflammatory pathways are connected, possibly through a mechano-protective effect against downstream degeneration from TNF stimulation^23^ Similarly, AC chondrocytes from patients suffering from rheumatoid arthritis or osteoarthritis have been shown to respond differentially to mechanical stimuli in comparison to chondrocytes from healthy AC^24–27^ However, little is known about CEP mechanobiology and how it might be altered in a pro-inflammatory, catabolic environment. As experimental and in situ evidence are challenging to acquire and/or interpret, physics-based computer models have helped to apprehend possible consequences of CEP degradation at the disc organ level^5^ Regulatory network models (RNM) have also been proposed to explore shifts in cartilaginous cell activity under various mechano-chemical environments^28,29^ Remarkably, these models easily allow integration of biological knowledge and data, to support interpretation of experimental models.

As per experimental models, agarose has been widely used for culture of articular chondrocytes as it offers a uniform, uncharged 3D environment that can withstand mechanical loading and maintain chondrocyte phenotype over extended periods. Studies have shown that in agarose, articular chondrocytes can synthesize ECM which is further enhanced through dynamic compression^30–32^ Further, after long-term cultures (~ one month), articular chondrocyte-seeded hydrogels can reach properties close to one-quarter those of AC tissues,^30^ offering a promising tissue-engineered 3D culture model for cartilaginous tissues. However, while extensive research has been conducted on articular chondrocytes in agarose^30–32^ at the time of this publication, no study exists using 3D culture of CEP cells in agarose. However, the CEP is a unique tissue distinct from AC, BEP, and IVD tissues, thus it is critical to characterize the CEP and CEP cells^2^.

In this study, it was hypothesized that dynamic compression would be sufficient to induce anabolism in CEP cells, while stimulation with pro-inflammatory cytokines would induce catabolism. To test this hypothesis, CEP cell-seeded agarose hydrogels were cultured for two weeks under dynamic compression and stimulation with TNF and evaluated through gene expression, secretome quantification, cell metabolism, and glycosaminoglycan (GAG) production analyses. Experimental results were compared to in silico RNM which provided auxiliary and exploratory information on the mechanobiological signaling pathways.

## Results

### Cell viability did not change during culture

**Fig. 1:**
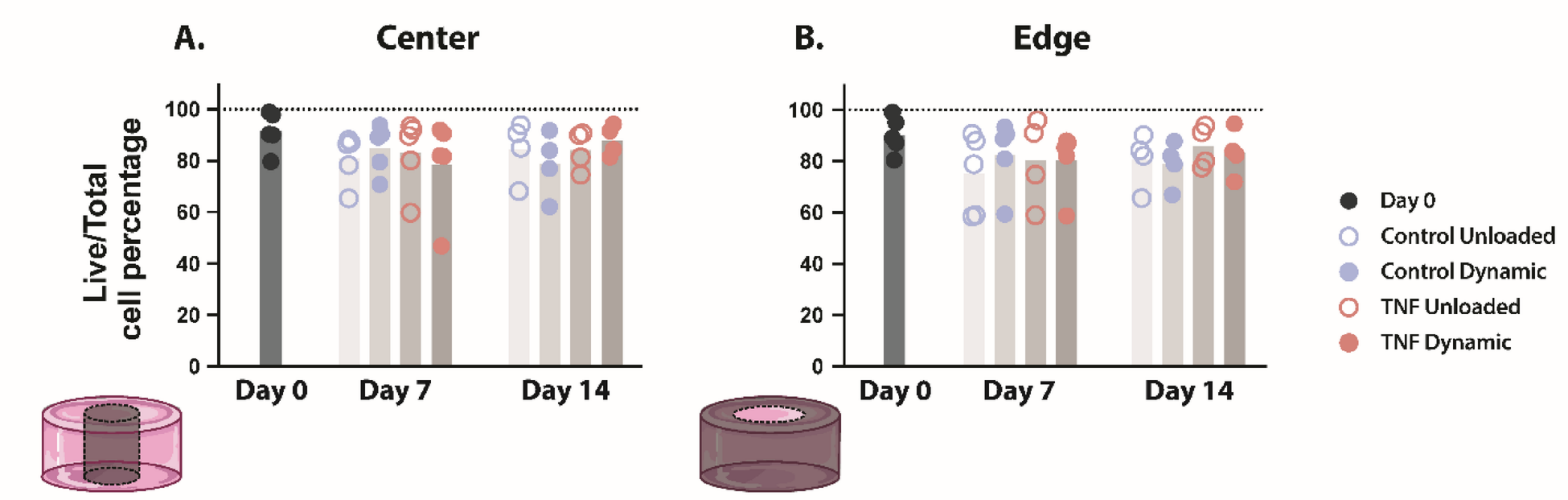
Cell viability of human CEP cells remained stable during culture. Cell viability of CEP cells in agarose at the (**a**) center and (**b**) edge of the hydrogel. Shown are the medians, *n* = 4–5.

To verify 2% agarose as a viable hydrogel for 3D culture of CEP cells, live and dead cells were quantified on days 0, 7, and 14 at both the center (Fig. 1a) and edge (Fig. 1b) of the hydrogels. Initial cell viability at day 0 ranged from 79.6 to 99.0%, with a median of 90.3% and 89.1% at the center and edge, respectively. There was no significant change in cell viability in any condition or location after 7 or 14 days of culture. However, while median cell viability remained relatively constant over time for all conditions (between 78.8 and 89.7%), cell viability showed greater variability between donors. Hydrogels in the unloaded control condition showed a cell viability ranging from 58.5 to 93.9%, while that of the dynamically compressed control condition was 59.4–93.9%. Similarly, cell viability in the unloaded TNF-treated condition ranged from 58.9 to 96.0%, while that of the dynamically compressed TNF-treated condition was 46.9–94.7%. Remarkably, minimum cell viabilities for each condition were consistently measured for the same donor, conveying that donor had a larger effect on cell viability than loading or TNF treatment.

### Cell metabolism and GAG/DNA content remained constant

**Fig. 2:**
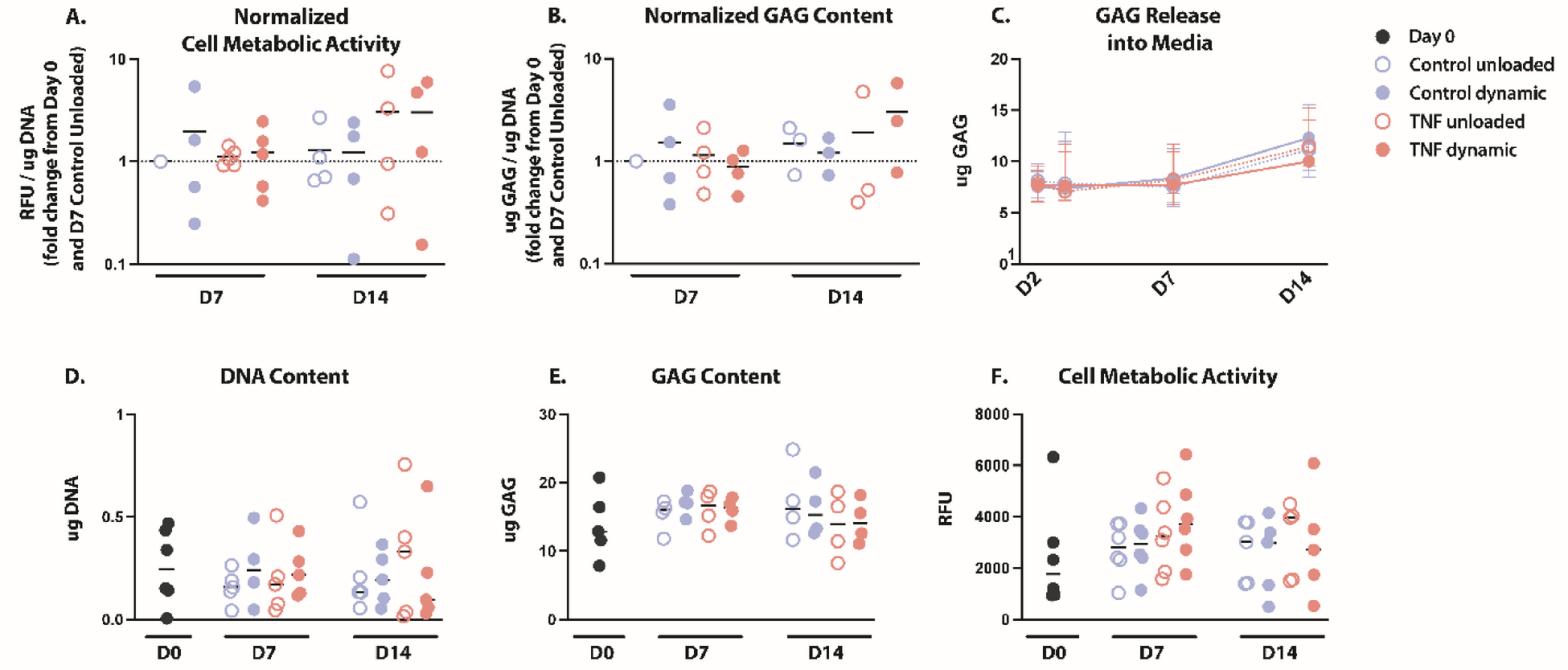
Cell metabolism and GAG/DNA content in human CEP cells remained constant over time. (**a**) Cell metabolic activity normalized to corresponding DNA content, shown as the fold change from Day 0 and the Day 7 control unloaded condition. (**b**) GAG content normalized to corresponding DNA content, shown as the fold change from Day 0 and the Day 7 control unloaded condition. (**c**) GAG content measured in the secretome throughout culture. (**d**) DNA content in ug of CEP cells in agarose after 7 and 14 days in culture. (**e**) GAG content in ug of CEP cells in agarose after 7 and 14 days in culture. (**f**) Cell metabolic activity of CEP cells in agarose after 7 and 14 days in culture. Shown are the medians, *n* = 4–5.

Cell metabolism (Fig. 2a, f) and GAG production (Fig. 2b, e) and release into the media (Fig. 2c) were not significantly different after culture for any condition. GAG content stayed relatively constant at ~ 15 µg throughout the duration of the experiment but increased from a median of 12.891 ug to between 13.913 and 16.056 ug after 7 or 14 days culture (Fig. 2e). DNA content was not significantly different between groups, but trended towards a decrease after 7 and 14 days culture (Fig. 2d). When normalizing GAG content by DNA content, no significant differences remained. However, when compared to Days 0 and 7, GAG/DNA content was decreased in unloaded hydrogels, TNF treated hydrogels and increased in dynamically loaded TNF treated hydrogels after 14 days (Fig. 2b). GAG release into the media stayed constant for the first 7 days however was showed a trend toward an increase on day 14 in all conditions except for the unloaded control (dynamic control: *p* = 0.06; dynamic TNF: *p* = 0.06; unloaded TNF: *p* = 0.06) (Fig. 2c). Interestingly, cell metabolic activity showed a nonsignificant increase after 14 days when normalized to DNA content, but not after 7 days, in TNF-treated hydrogels when compared to controls (Fig. 2a).

### Gene expression is altered due to TNF stimulation but not mechanical loading

**Fig. 3:**
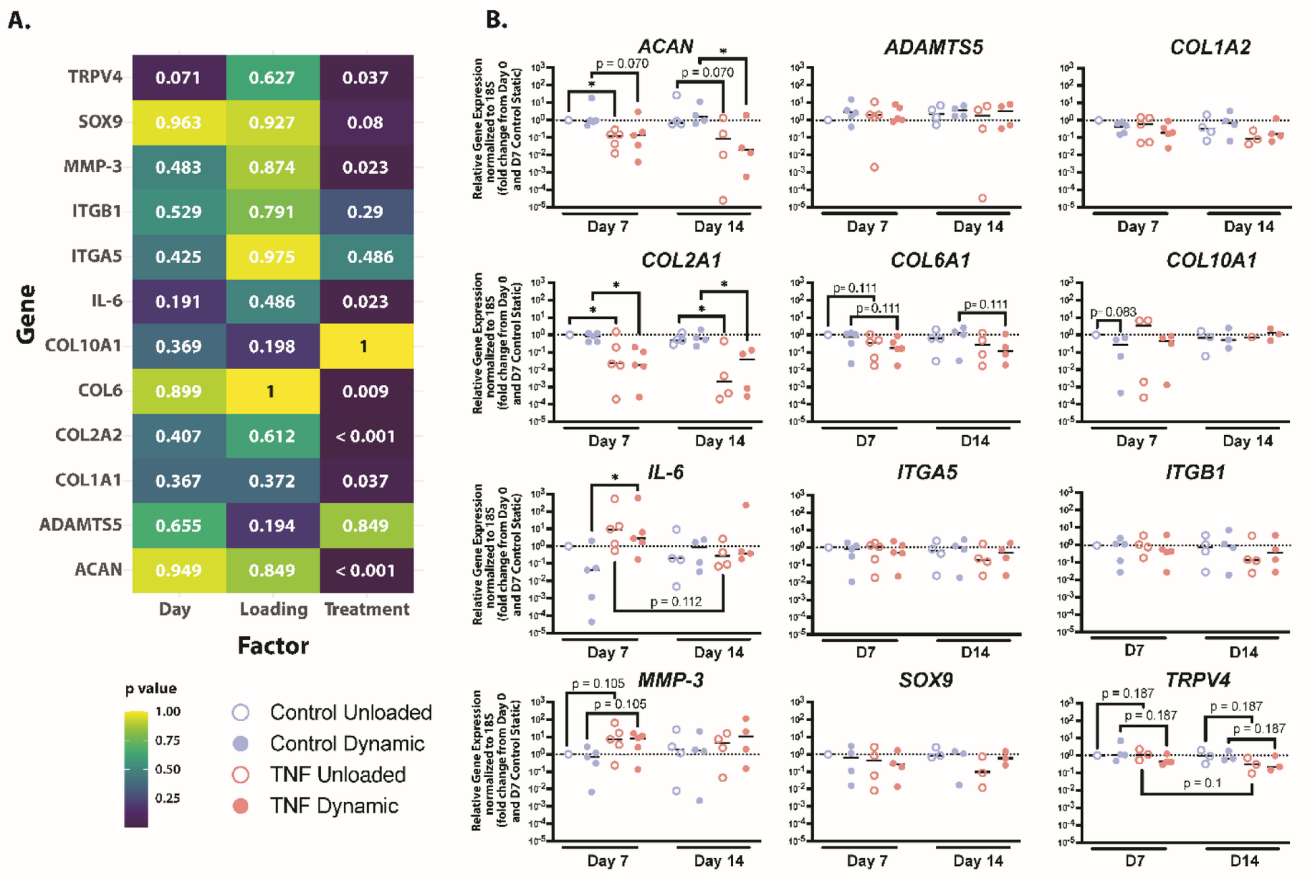
Gene expression shifts to catabolic phenotype after TNF stimulation in human CEP cells. (**a**) Heatmap of the *p* values, determined through Kruskal Wallis statistical analysis, for the gene expression results based on day, loading, and treatment. (**b**) Relative gene expression data for genes *ACAN*, *ADAMTS5*, *COL2A1*, *COL6A1*, *COL1A2*,* COL10A1*,* IL-6*,* ITGA5*,* ITGB1*,* MMP3*,* SOX9*,* and TRPV4* shown as the fold change from Day 0 and the Day 7 control unloaded condition. Shown are the medians, *n* = 4–5. *p < 0.05.

To check the cellular response to TNF treatment and dynamic compression, quantitative polymerase chain reaction (qPCR) was done to quantify gene expression in key anabolic, catabolic, and mechanobiological markers. While dynamic compression or time had no significant effect on any gene expression, treatment with 10 ng/mL TNF had a significant effect on *ACAN*, *COL2A1*, *COL6A1*, *IL6*, and *MMP-3* expression (Fig. 3a, b).

After 7 and 14 days of culture TNF-stimulated hydrogels trended towards decreased expression of genes coding for proteoglycan and collagens production (Fig. 3b). Specifically, unloaded and dynamically loaded hydrogels after 7 and 14 days showed significantly less *COL2A1* expression when stimulated with TNF compared to controls (*p* = 0.017). *ACAN* expression significantly decreased in TNF-stimulated unloaded hydrogels after 7 days (*p* = 0.025) and in TNF-stimulated dynamically compressed hydrogels after 14 days (*p* = 0.044). TNF-stimulated dynamically loaded hydrogels after 7 days and unloaded hydrogels after 14 days were not significant (*p* = 0.07 for both) but trended towards decreased expression. Similarly, PCM marker *COL6A1* exhibited a trend (*p* = 0.111) towards decreased expression in TNF-stimulated hydrogels compared to controls. *COL1A2* and *SOX9* were not significantly different between any condition.

*IL-6* and *MMP-3* were upregulated after TNF stimulation for 7 days, however this effect disappeared after 14 days. Specifically, *IL-6* expression was significantly increased in TNF-stimulated dynamically compressed hydrogels compared to dynamically compressed controls (*p* = 0.012). Additionally, TNF-stimulated unloaded hydrogels trended towards a decrease in expression of *IL-6* from 7 to 14 days (*p* = 0.112). While *MMP-3* variations were not significant, trends towards increased expression were detected in TNF-stimulated unloaded and dynamically loaded hydrogels after 7 days of culture (*p* = 0.105). Calcification marker *COL10A1*,^33^ was not significantly altered in any condition, however the control dynamically loaded hydrogels showed a trend (*p* = 0.083) toward decreased *COL10A1* expression in comparison to control unloaded hydrogels. *ADAMTS5* expression was not significantly different in any condition.

The expressions of several mechanosensors were also measured to evaluate the mechanobiological effects of TNF stimulation and dynamic compression at the gene level (Fig. 3b). *TRPV4* is a Ca^2+^-permeable osmo-mechanical-TRP channel^34^ However, neither dynamic loading nor stimulation with TNF produced a significant difference in *TRPV4* expression after 7 or 14 days of culture in agarose, although TNF stimulation did show a trend towards decreased expression (*p* = 0.187). Similarly, gene expression of integrins α5 and β1 were not significantly altered by TNF stimulation or dynamic compression. Overall, gene expression of the cultured cells remained largely unresponsive to mechanical loads.

### TNF promotes secretion of pro-inflammatory factors in CEP cells

**Fig. 4:**
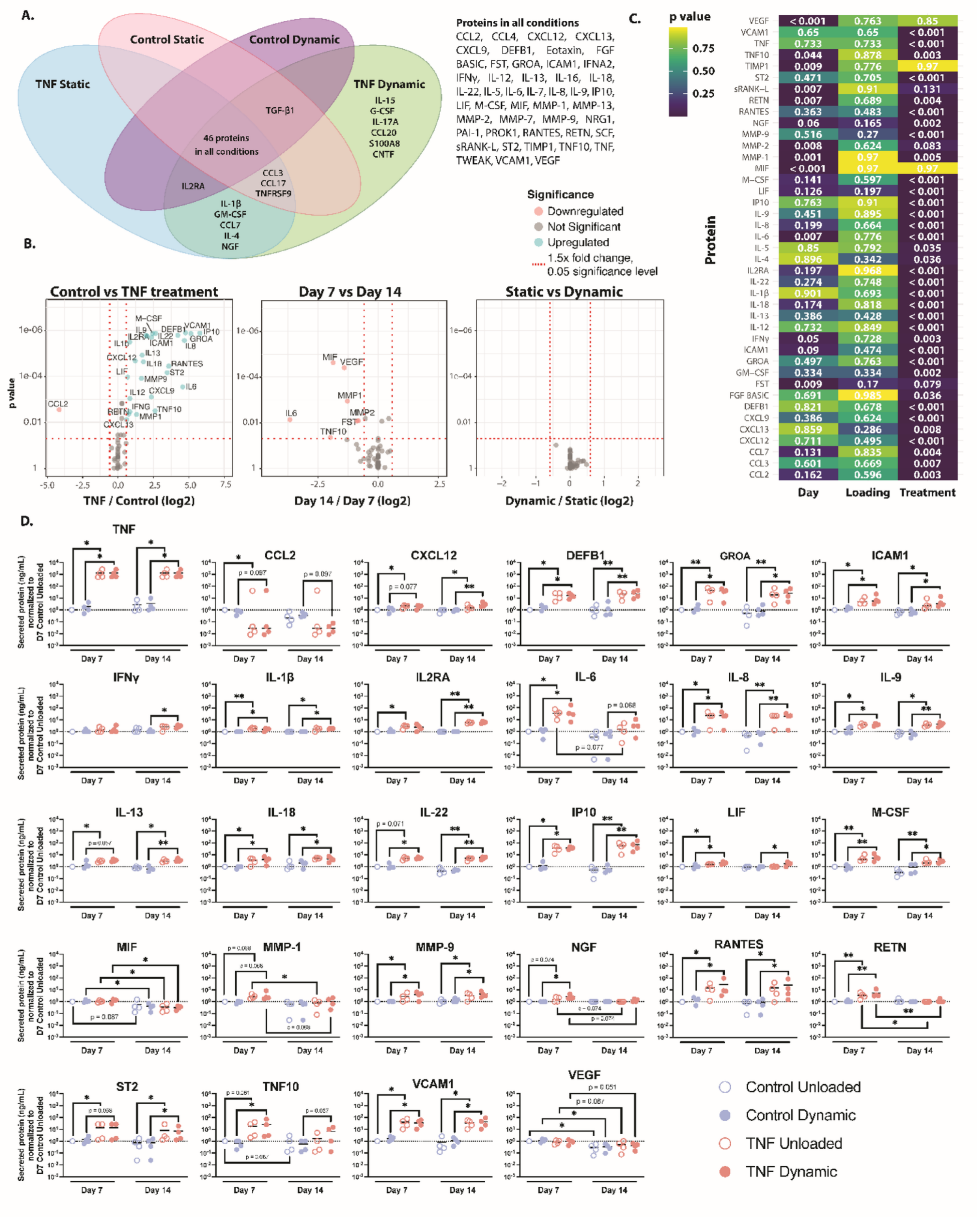
TNF promotes secretion of pro-inflammatory factors in human CEP cells. (**a**) Venn diagram showing the proteins detected in the secretome of the control unloaded (pink), control dynamic (purple), TNF unloaded (blue), and TNF dynamic (green) conditions. Proteins detected in all four categories are listed next to the diagram. (**b**) Volcano plots showing proteins that were found to be statistically significant (*p* > 0.05) and a fold change > 1.5x comparing control vs. TNF treatment, Day 7 versus Day 14, and unloaded versus dynamic compression. Statistics were done by first normalizing to the control unloaded condition, followed by Kruskal Wallis analysis. (**c**) Heatmap of the *p* values, determined through Kruskal Wallis statistical analysis, for the protein secretion results based on day, loading, and treatment. Only proteins which were found significant in the volcano plots are shown in the figure. (**d**) Secreted protein data (ng/ml) normalized to the Day 7 control unloaded condition. Proteins are listed in alphabetical order. Only proteins with statistical differences are shown in the figure, including TNF, CCL2, CXCL12, DEFB1, GROA, ICAM1, IFN-γ, IL-1β, IL-2Ra, IL-6, IL-8, IL-9, IL-13, IL-18, IL-22, IP10, LIF, M-CSF, MIF, MMP-1, MMP-9, NGF, RANTES, RETN, ST2, TNF10, VCAM1, and VEGF. Shown are the medians, *n* = 4–5. *p < 0.05, **p < 0.01.

Out of 73 proteins tested, 46 proteins were detected in all conditions (Fig. 4a). 14 proteins were detected at low levels in the uncultured media sample, chemokine (C-X-C motif) ligand (CXCL) 9, fibroblast growth factor (FGF) BASIC, growth regulated protein alpha (GROA), intercellular adhesion molecule 1 (ICAM1), IL-18, IL-5, IL-8, MMP-2, plasminogen activator inhibitor-1 (PAI-1), stem cell factor (SCF), soluble RANK ligand (sRANK-L), TNF10, TNF, and vascular endothelial growth factor (VEGF). Additionally, five proteins, IL-1β, granulocyte macrophage colony-stimulating factor (GM-CSF), chemokine (C-C motif) ligand (CCL) 7, IL-4, and nerve growth factor (NGF), were found in the TNF-treated secretome, while six more proteins, IL-15, granulocyte CSF (G-CSF), IL-17 A, CCL20, S100 calcium-binding protein A8 (S100A8), and ciliary neurotrophic factor (CNTF), were found only in the TNF-treated, dynamically compressed hydrogel secretome. IL-2 receptor alpha (IL-2RA) was present in all conditions except for the control, unloaded hydrogel secretome. Similarly, the proteins CCL3, CCL17, and TNF receptor superfamily member 9 (TNFRSF9) were secreted in all but the control, dynamically compressed hydrogels. Transforming growth factor (TGF)-β1 was only not secreted in the TNF-treated, unloaded hydrogels.

Dynamic compression had no significant effect on the secretome when compared to unloaded hydrogels. TNF treatment had a much greater effect on the secretome than duration of culture or dynamic compression (Fig. 4b, c,d). Specifically, TNF increased secretion of pro-inflammatory factors, including IL-1β, RANTES (or CCL5), IL-6, IL-8, and ST2 (or IL-1 receptor-like 1). Other interleukins were upregulated in the secretome of TNF treated hydrogels, including IL-9, IL-13, IL-18, and IL-22. Interestingly, IL-4 was not different between any condition and was not detected in the secretome of control, unloaded or dynamically loaded hydrogels. IL-10, an anti-inflammatory cytokine, was not detected either, in any condition. Inflammatory mediators ICAM-1 and vascular adhesion molecule 1 (VCAM-1)^35^ were also highly upregulated, 2.38- and 4.78-fold times greater than the control, respectively, in the secretome following TNF stimulation. M-CSF and MMP-9 secretion was significantly increased in TNF-treated hydrogels relative to their respective controls. Chemokines GROA, CXCL9, 12, and 13 were also found secreted higher after TNF stimulation relative to controls. Further, interferon gamma (IFN-γ) and IFN-γ-induced protein 10 (IP10) were increased following TNF stimulation. IFN-γ was only significantly upregulated in dynamically loaded hydrogels after 14 days (*p* = 0.012), while IP10 was significantly upregulated greater than 5x relative to controls at 7 and 14 days (*p* = 0.003 for both on day 14, *p* = 0.045 and 0.036 for day 7 unloaded and dynamic hydrogels, respectively). Beta-defensin 1, DEFB1 was secreted 4.23x higher in TNF treated conditions than controls. Notably, the only cytokine whose secretion was downregulated by TNF stimulation was CCL2, also known as monocyte chemoattractant protein 1 (MCP-1). This downregulation was only significant in the unloaded hydrogels after 7 days (*p* = 0.044), however the trend was seen in dynamically loaded hydrogels on days 7 and 14 (*p* = 0.097 for both). Regarding phosphorylated protein results, TNF stimulation increased transcription factor NFκB content between 2-14x, however more biological replicates are needed to evaluate significance (see Supplementary Figure S1).

### Pain, neurotrophic and angiogenic factor secretion decreases over time in agarose culture

Some proteins, particularly angiogenic and neurotrophic factors, were secreted significantly less over time, conveying a time-dependent response to TNF stimulation, with no changes due to dynamic compression (Fig. 4b, c,d). Resistin (RETN), an adipose-derived hormone, was significantly upregulated by TNF treatment after 7 days of culture (*p* = 0.007 in both unloaded and dynamically loaded hydrogels). However, this behavior disappears after 14 days, where TNF treated hydrogels return to control levels of RETN secretion. While VEGF, a key regulator of angiogenesis, secretion was not significantly different amongst conditions, its secretion significantly decreased after 14 days relative to its level after 7 days. NGF, a neurotrophic factor, secretion was only significantly different between TNF treated, dynamically compressed hydrogels relative to dynamically compressed controls after 7 days culture. However, after 14 days any differences in NGF secretion disappeared. Secretion of IL-6 was highly upregulated in TNF treated hydrogels relative to controls on day 7 (*p* = 0.029 for both unloaded and dynamic conditions), however this pattern did not hold for the longer 14 day culture time. Macrophage inhibitory factor (MIF) was also found to be significantly decreased over time in all conditions (*p* = 0.021, *p* = 0.021, *p* = 0.013 for control dynamic, TNF unloaded, and TNF dynamic, respectively) except for the control unloaded hydrogels (*p* = 0.087).

MMP-1 secretion was increased in TNF treated hydrogels on day 7 for both unloaded (*p* = 0.068) and dynamically loaded (*p* = 0.068) conditions. However, MMP-1 secretion decreased in the TNF conditions to match controls after 14 days (*p* = 0.031 and *p* = 0.068 for unloaded and dynamically loaded hydrogels, respectively). Follistatin (FST) and MMP-2 secretion were significantly decreased collectively from 7 to 14 days, but were not significant for specific conditions. However, the trend towards a decrease in FST, which can be anti-inflammatory and help to maintain ECM integrity, was stronger in control hydrogels (*p* = 0.105) in comparison to TNF treated hydrogels (*p* = 0.237 and *p* = 0.186 for unloaded and dynamically loaded conditions, respectively). Similarly, TNF10 secretion was significantly greater in TNF treated, dynamically loaded hydrogels relative to dynamically loaded controls (*p* = 0.03) and collectively decreased secretion after 14 days relative to 7 days but did not decrease significantly in any specific condition.

#### Semi-quantitative comparison shows low deviation between RNM and experiment results

**Fig. 5:**
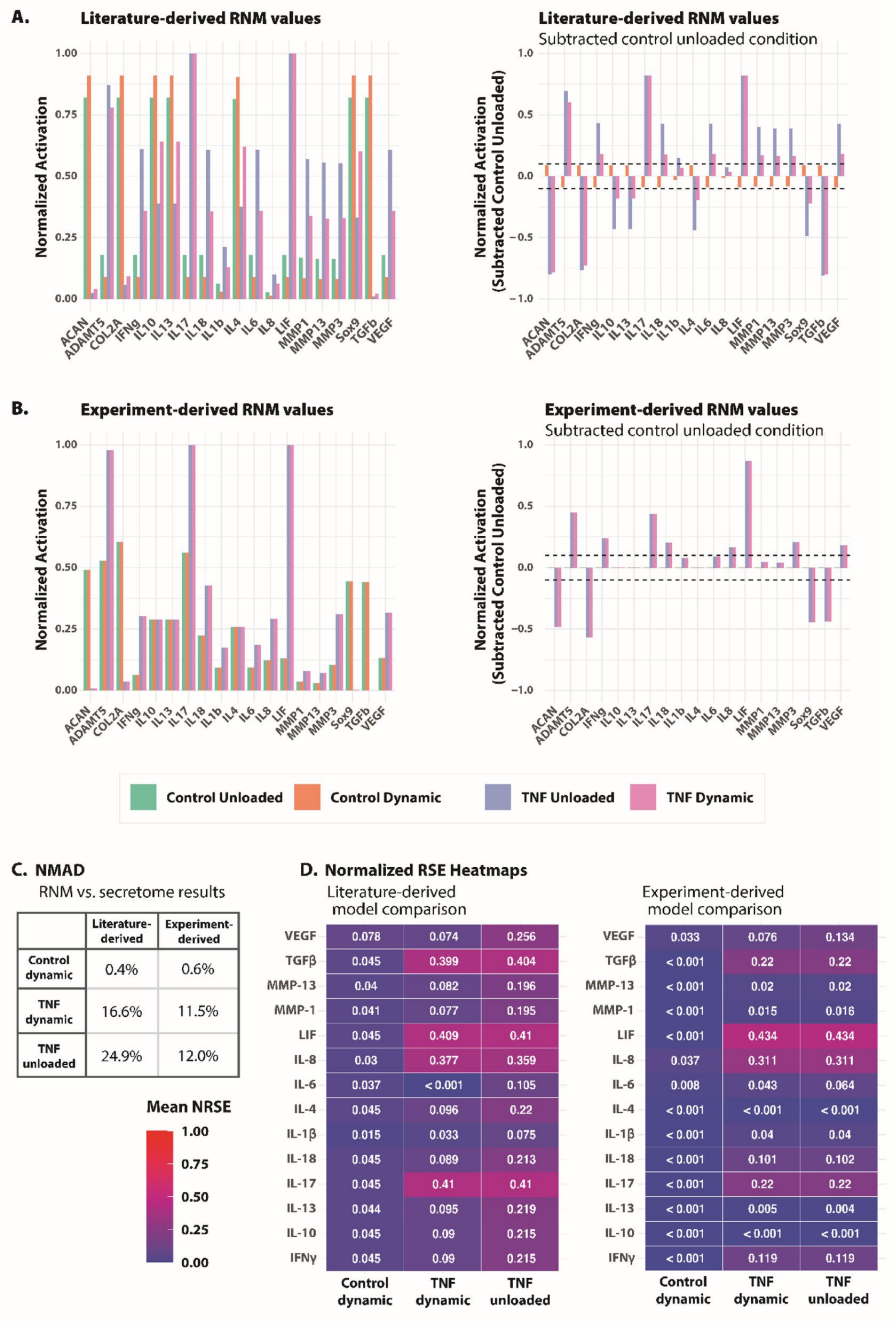
Semi-quantitative comparison of RNM and experiment results.

The literature-derived RNM predicted only small changes (< 10%) between the unloaded and dynamic conditions (Table S1), however anabolic factors ACAN, COL2A1, SOX9, and TGF-β were predicted to increase under dynamic compression, while catabolic factors ADAMTS5, IFNγ, IL-6, IL-17, IL-18, LIF, VEGF, and MMPs − 1, -3, and − 13 were predicted to decrease (Fig. 5a). Simulated TNF stimulation predicted decreased anabolic factors (ACAN, COL2A1, SOX9, and TGF-β) and increased catabolic factors (ADAMTS5, IFNγ, IL-1β, IL-6, IL-17, IL-18, LIF, MMP-3, and VEGF). Literature-derived simulations of TNF stimulation also saw an increase in MMPs − 1 and − 13, while experiment-derived simulations of TNF stimulation showed an increase in IL-8. Further, literature-derived simulations found that simulated dynamic compression decreased these catabolic factors that were increased from TNF relative to the simulated unloaded condition.

Protein activation levels from the RNM simulations for both literature-derived and experiment-derived approaches were semi-quantitatively compared to protein secretion values using normalized mean absolute deviation (NMAD) to measure the cumulative error between the predicted and the experimental datasets, and the normalized root squared error (NRSE) following methods established by Segarra-Queralt et al.^36^ (Fig. 5). NMAD found lower deviation in control dynamic simulations (0.4–0.6%) than TNF-stimulated conditions (11.5–24.9%) (Fig. 5c). Experiment-derived RNM simulations had less deviation in the TNF-stimulated conditions from secretome results than literature-based simulations for both unloaded (12% and 24.9%, respectively) and dynamic conditions (11.5% and 16.6%, respectively). NRSE calculated for each protein conveyed that error within TNF-stimulated conditions primarily occurred due to incongruent responses of four proteins: TGF-β, LIF, IL-8, and IL-17 (Fig. 5b, c). Interestingly, RNM simulations could lead to different steady states contingent on if initial conditions leaned anabolic or catabolic. For experimental-derived RNM simulations, 56% of the control cases and 100% of TNF-stimulated cases led to catabolic steady states. For literature-derived RNM simulations, 82% of control unloaded cases and 91% of control dynamic cases led to anabolic steady states. Similar to experiment-derived simulations, 100% of TNF-stimulated literature-derived cases led to catabolic steady states.

## Discussion

This study confirmed the hypothesis that TNF is sufficient to induce a catabolic response in human CEP cells through downregulation of anabolic gene expression and upregulation of pro-inflammatory proteins associated with immune cell recruitment,^35^ herniated discs^37,38^ bacteria inhibition,^39^ pain,^38,40,41^ and Modic changes^4,42^ However, dynamic compression did not lead to anabolism in the CEP cells as predicted. The decrease in *ACAN*, *COL2A1*, and *COL6A1* and increase in *MMP-3* gene expression following TNF stimulation aligns with catabolic changes to the ECM and PCM. Reduced GAGs and *COL2A1* lead to reduced hydration, impairing the ability of the CEP to withstand loading and transport essential nutrients, rendering it more susceptible to cracks or fissures that bring inflammation, blood vessel and nerve ingrowth, and painful disc degeneration. Further, increased *IL-6* expression supports the induction of a pain phenotype. Likewise, protein secretion corroborated gene expression data, with TNF-stimulated hydrogels secreting significantly more proteins that have been implicated in disc degeneration and chronic low back pain, IL-1β, RANTES, IL-6, IL-8, and ST2^40,41^ Similarly, TNF stimulation led to increased secretion of factors which have been correlated to herniation, IFN-γ^37^ and IP10^38^, alongside chemokines that are upregulated in spinal cord injury and arthritis, GROA, CXCL9, 12, and 13,^43^ and inflammatory mediators ICAM-1 and VCAM-1^35^ While NFκB phosphorylation was increased with TNF stimulation, ERK1 phosphorylation was not detected, despite prior research of ERK1/2 involvement in TNF signaling in IVD NP cells^44^.

Notably, DEFB1 was highly secreted by TNF-stimulated hydrogels. Despite the role of DEFB1 in immune defense and antimicrobial activity,^39^ it has not yet been explored in the IVD. Rajasekaran et al.^45^ found defensins in degenerate, but not in healthy discs, suggesting that subclinical infection may play a role in IVD degeneration. In AC, DEFB1 and IP10 (also called CXCL10), whose secretion was also upregulated following TNF stimulation, were identified to contribute to the pro-inflammatory response of chondrocytes and act as key, but underreported players in osteoarthritis^46,47^ DEFB1 signaling occurs through TLR4 signaling,^46,48^ whose expression in the CEP has been suggested to play a detrimental role in endplate degradation^17^ As the CEP is the IVD’s first line of defense against bacterial infection, it is noteworthy that an antimicrobial factor such as DEFB1 is secreted under pro-inflammatory stimulation as it could indicate an immunomodulatory role in CEP cells and could be probed further as a novel therapeutic target.

An unexpected response was the decreased secretion of CCL2 in response to TNF stimulation. CCL2, a chemoattractant for monocytes and basophils,^49^ is typically upregulated in response to TNF or other inflammatory stimulation^50^ but this response is time-dependent^51^ and has been shown to decrease with age^52^ However, De Luca et al.^49^ showed that while pro-inflammatory cytokine IL-1β upregulated CCL2 gene expression, protein release of CCL2 was decreased in endplate cells, consistent with our findings with TNF stimulation. Similarly, Nakawaki et al.^53^ found CCL2 protein to be elevated for the initial three days following TNF stimulation of mice IVDs but returned to basal levels after seven days. The role of CCL2 in AC has also been a source of contention, with some studies suggesting CCL2 deficiency protects against osteoarthritis,^54^ while other studies claiming CCL2 is necessary for cartilage regeneration^55^ Accordingly, CCL2 might be decreased due to a negative feedback loop^49,53^ and thus timing is crucial when considering its role in degeneration. Besides results achieved for CCL2, the increase of VCAM-1 and ICAM-1 upon TNF stimulation are worth mentioning, as both proteins are involved in the adhesion of leukocytes. While the stimulation of VCAM-1 and ICAM-1 by TNF is commonly reported in the literature,^35^ the implication for CEP cells indicates the potential of interaction with the innate immune system through the peripheral microvasculature.

Protein secretion of CEP cells also exhibited a time-dependent response to stimulation with TNF. In particular, secretion of angiogenic and neurotrophic proteins decreased over time. VEGF and RETN, both of which stimulate angiogenesis and have been implicated in metabolic disorders^56,57^ Notably, NGF, which is associated to nociceptive nerve ingrowth and painful IVD degeneration,^58^ was increased after 7 days, corresponding to prior studies showing NGF increase following TNF and IL-1β stimulation^59^ However, after 14 days of culture, NGF secretion returned to control levels. This same trend was also seen in MMP-1 and IL-6 secretion, both of which have been found in degenerative IVDs^60^ Interestingly, MIF secretion also decreased over time, however, it was unaffected by TNF stimulation. MIF has been noted to be upregulated in CEP cells following TNF stimulation,^61^ and has been suggested to be a part of a positive inflammatory feedback loop that escalates inflammation in MC^2^ Overall, the decreased secretion of angiogenic and neurotrophic proteins suggests that CEP cells can adapt to a prolonged inflammatory environment and that there might be a mechanism which works to prevent blood vessel and nerve ingrowth into the disc.

Despite prior findings of GAG production and chondrogenesis in dynamically loaded AC chondrocytes in 2% agarose,^30–32^ dynamic compression did not have any significant effects in this study. Interestingly, GAG release into the media displayed an insignificant increase in all conditions on day 14 relative to initial GAG release. In seeing these changes start to occur at the end of culture, it is possible that longer timepoints would be needed to see significant changes in GAG content^31^ Additionally, other studies seeing an increase in GAG content from chondrocytes seeded in agarose used serum throughout the entirety of the culture,^31,62^ while our study used serum-free media to avoid interfering with the results from adding TNF, which could have decreased the amount of GAGs produced. The 7% strain on the hydrogels is within the ≤ 20% anabolic physiological range for cell strain in cartilage^63,64^ However, this strain might not be enough to affect CEP cells in 3D culture. While 7% strain was chosen to prevent cracks or breakage within the hydrogel, other studies have used higher strains of 10%,^31,66^ 15%,^65^ and 20%^67^ and seen more significant changes. However, Jutila et al.^67^ found significant changes in metabolites in chondrocyte-seeded agarose hydrogels following dynamic compression of 4.5 to 5.5% strain but suggested that 2% agarose may be closer to the stiffness of osteoarthritic PCM and recommended > 4.5% agarose for modeling healthy cartilage. Agarose has been considered the gold standard in cartilage biomaterials because it is biocompatible and preserves a round cell morphology while remaining strong enough to withstand compressive loading^30,68^ However, agarose is not ideal for mechanotransduction as it does not allow for mechanosensors, particularly integrins, to interact with the ECM and transmit the mechanical signals to the cells^66^ Studies have had success in implementing a pre-culture period to allow the formation of a PCM^68–70^ However, five days did not make a difference for CEP cells in 2% agarose, although *COL6A1* was expressed in all hydrogels suggesting that a PCM might have been produced. Accordingly, an alternate explanation could be that the produced PCM was stiffer than the 2% agarose, and thus the dynamic compression signals were not enough to reach the cells. Interestingly, TNF did decrease *COL6A1* expression, suggesting that a pro-inflammatory microenvironment affects PCM production. According to the RNM simulations, should the mechanosensors, specifically α5β1 and αvβ3 integrins that have been shown as expressed in chondrocytes following dynamic compression,^71^ not be activated in the hydrogels, then the expected downstream downregulation of pro-inflammatory pathways and upregulation of GAG production could not occur. Gene expression data regarding *ITGA5* and *ITGB1* support this, as expression did not change with dynamic compression. Thus, it is likely that the mechanical signals were not effectively sensed by the cells in the agarose gels, beyond basal cell-material /matrix interaction. The pro-anabolic effects of α_5_β_1_ and α_v_β_3_ depend on these integrins’ capacity to bind to functional fibronectin sequences and the connection to the actin cytoskeleton. The RNM further corroborates the idea that integrin binding was lacking, as simulations clamping actin to zero were more comparable to experimental results. Accordingly, future studies on mechanotransduction in CEP using agarose should blend other biomaterials that support interaction between the ECM and cell mechanosensors such as collagen,^66,72^ laminin,^73^ hyaluronic acid,^72^ and methacrylated gelatin^65^.

While the RNM is based on AC chondrocytes, predictions showed low (< 25%) deviation from experimental results, conveying a similar response to inflammation and dynamic compression between AC chondrocytes and CEP cells and highlighting protein interactions that should be further investigated. Interestingly, the literature-derived RNM predicted only small changes (< 10%) between the unloaded and dynamic conditions, although the dynamic stimulation was 9% more likely than unloaded hydrogels to produce an anabolic steady state. Notably, IL-4 plays a key role in the RNM and is induced from healthy physio-osmotic conditions and α_5_β_1_ activation while low (< 0.2585) IL-4 activation triggers catabolism^28^ It is possible that the critical point where IL-4 switches from a catabolic to anabolic state is below the threshold for detection within the Luminex system and that more sensitive assays are required. However, within the secretome IL-4 was only detected in TNF-stimulated conditions, suggesting it could play a more catabolic role in CEP cells.

Semi-quantitative comparison between the secretome and RNM outputs also highlighted the inaccuracy of TGF-β and IL-17, which were predicted by the RNM to be largely affected by TNF despite experimental results indicating their secretion was unaffected. Thus, interactions of TGF-β and IL-17 with TNF seen in the RNM were not found to be present in CEP cells. Simulations also predicted SOX9 to decrease with TNF stimulation, however no significant changes were seen at the transcript level in experiment data. Additionally, the RNM predicted decreased activation in TNF-stimulated conditions in contrast to measured secretion of IL-13 which was significantly increased in TNF-stimulated conditions. This suggests IL-13 might be secreted to compensate for the pro-inflammatory effects of TNF or that it plays a more catabolic role within the CEP. Thus, the regulation and interactions of IL-13 should be further investigated and added to the RNM for more robust predictions of chondrocyte response to pro-inflammatory stimuli. Notably, the RNM consistently predicted aggrecanase-2 ADAMTS5 to increase after stimulation with TNF, ADAMTS5 was not found to change at the transcript level following TNF stimulation experimentally. In contrast, prior research found ADAMTS5 and TNF upregulated in degenerative CEP tissue and bovine endplate cells,^74^ suggesting that these interactions in human CEP cells in agarose are absent.

## Conclusions and limitations

In conclusion, agarose is sufficient for investigation of the effects of pro-inflammatory cytokine stimulation on human CEP cells, however it is not an ideal material for mechanotransduction. TNF stimulation induced a catabolic, time-dependent response in human CEP cells shown through downregulation of anabolic gene expression and increased secretion of pro-inflammatory proteins associated with herniated discs, bacteria inhibition, and pain. Dynamic compression at 7% strain had no effect on CEP cells seeded in 2% agarose, and thus future studies exploring mechanotransduction in the CEP should incorporate other biomaterials that support interaction between the ECM and cell mechanosensors.

Several limitations in the study should be noted. Activation of mechanosensors were only assessed at the transcript level and should though immunohistochemistry or Western blot would further confirm mechanosensor inactivation. The study was also limited by the capabilities of the bioreactor. The chambers needed 6 hydrogels for loading and frequency was constrained to 1.5 Hz, different than the physiological 1 Hz loading frequency^75^ Explant culture would have been ideal for this study but was not possible due to the difficulty of obtaining large enough intact CEP samples. Additionally, the tissue used to extract CEP cells came from trauma patients undergoing spinal surgery, as this was the closest to healthy possible. While the patients had no history of disc degeneration, the trauma itself could influence the cells. The proteomics analysis was limited to the detection of proteins included in the Luminex panel. Thus, additional proteins which were not assessed could also play a role in the CEP cellular response to TNF and not all nodes within the RNM could be evaluated experimentally. Phosphoproteins were limited to 2 donors and thus significant changes could not be evaluated. In particular, additional data regarding focal adhesion kinase (FAK) phosphorylation would further demonstrate the lack of mechanotransduction in the agarose hydrogels. The RNM used for comparison was built for AC chondrocytes, and thus is not optimized for CEP cells. It also does not account for time, consequently, changes between days 7 and 14 could not be simulated. However, no network model specific to the CEP exists at the time of publication and when CEP parameters are unknown, AC is often used as a substitute. Therefore, comparison to an AC chondrocyte RNM can provide a general overview of cell signaling in the CEP and further highlight proteins or cytokines that perform differently in the CEP and should be investigated further in future studies. Additionally, the RNM provides insights on integrin signaling when the connection to the ECM is broken by clamping nodes representing ECM proteins, such as actin, to zero.

## Methods

### Experimental model and study participant details

Human IVD tissues were collected from patients undergoing trauma surgery with informed consent of the patients or relatives and general ethical consent of the Insel University Hospital of Bern, Bern, Switzerland (donor characteristics are summarized in Table 1). The patients had no history of back pain or IVD degeneration.

**Table 1:** Donor characteristics summary of primary CEPC isolation.

Donor #	Gender	Age (years)	Location
1	Male	28	Th12/L1
2	Male	28	L1/L2
3*	Male	30	L2/L3
4	Male	30	L1/L2
5	Female	49	L1/L2
6	Female	41	Th12/L1

*Only used for day 0 and day 7 analysis due to limited number of cells following expansion.

### Human CEP cell isolation and expansion

Tissue fragments were morphologically separated carefully into NP, AF, and CEP tissues within 24 h of surgery. The CEP was identifiable as translucent cartilaginous tissue, much harder than the jelly-like NP or the fibrous AF and not porous as bone^6^ Primary human CEP cells were extracted from CEP tissue fragments by 1 h digestion in 1.9 mg/mL pronase (7 U/mg) (#10165921001, Roche Diagnostics, Basel, CH) followed by overnight digestion with collagenase II ((285 U/mg) Worthington, London, UK) in low-glucose (1 g/L) Dulbecco’s Modified Eagle Medium (LG-DMEM; #31600083; Gibco, Zug, CH) containing 10% *v*/*v* heat-inactivated fetal calf serum (FCS) (#F7524; Sigma-Aldrich, Buchs, CH), 1% *v*/*v* penicillin/streptomyacin (P/S) and 0.2% *v*/*v* primocin (#ant-pm; InvivoGen, San Diego, CA, USA, distributed by Lubioscience, inc., Lucerne, CH) on an orbital shaker at 37 °C. Following digestion, any remaining tissue was removed using 100 μm cell strainer filtration and cell viability and counting were determined via trypan blue. Following isolation, human CEP cells were expanded until passage three or four in monolayer in high-glucose (4.5 g/L) Dulbecco’s Modified Eagle Medium (HG-DMEM; #52100039; Gibco, Zug, CH) supplemented with 10% *v*/*v* heat-inactivated FCS and 1% *v*/*v* P/S and maintained at 37 °C in a humidified atmosphere containing 5% CO_2_ and normoxia and the medium was changed twice per week. Cells were never frozen prior the loading experiments.

### Hydrogel fabrication and culture

Agarose 2% wt/vol hydrogels were produced for culture of human CEP cells. In brief, agarose 4% wt/vol (2 g agarose powder; 50101, SeaPlaque low-gelling temperature, Lonza; dissolved in 50 mL 1X PBS) was autoclaved at 120 °C and stored at 60 °C until use. Cells were trypsinized and resuspended at a density of 15 × 10^6^ cells/mL in 1X PBS. The agarose solution was then mixed 1:1 with the cell suspension to obtain a final cell concentration of 7.5 × 10^6^ cells/mL^10^ in agarose 2% wt/vol. 85 µL of the mixture was then pipetted into silicon molds (Ø: 6 mm, height: 3 mm). Cell-agarose hydrogels were incubated at room temperature (RT) for 15 min and culture in LG-DMEM with 10% FBS for five days for phenotype recovery and to build the PCM^68–70^.

**Fig. 6:**
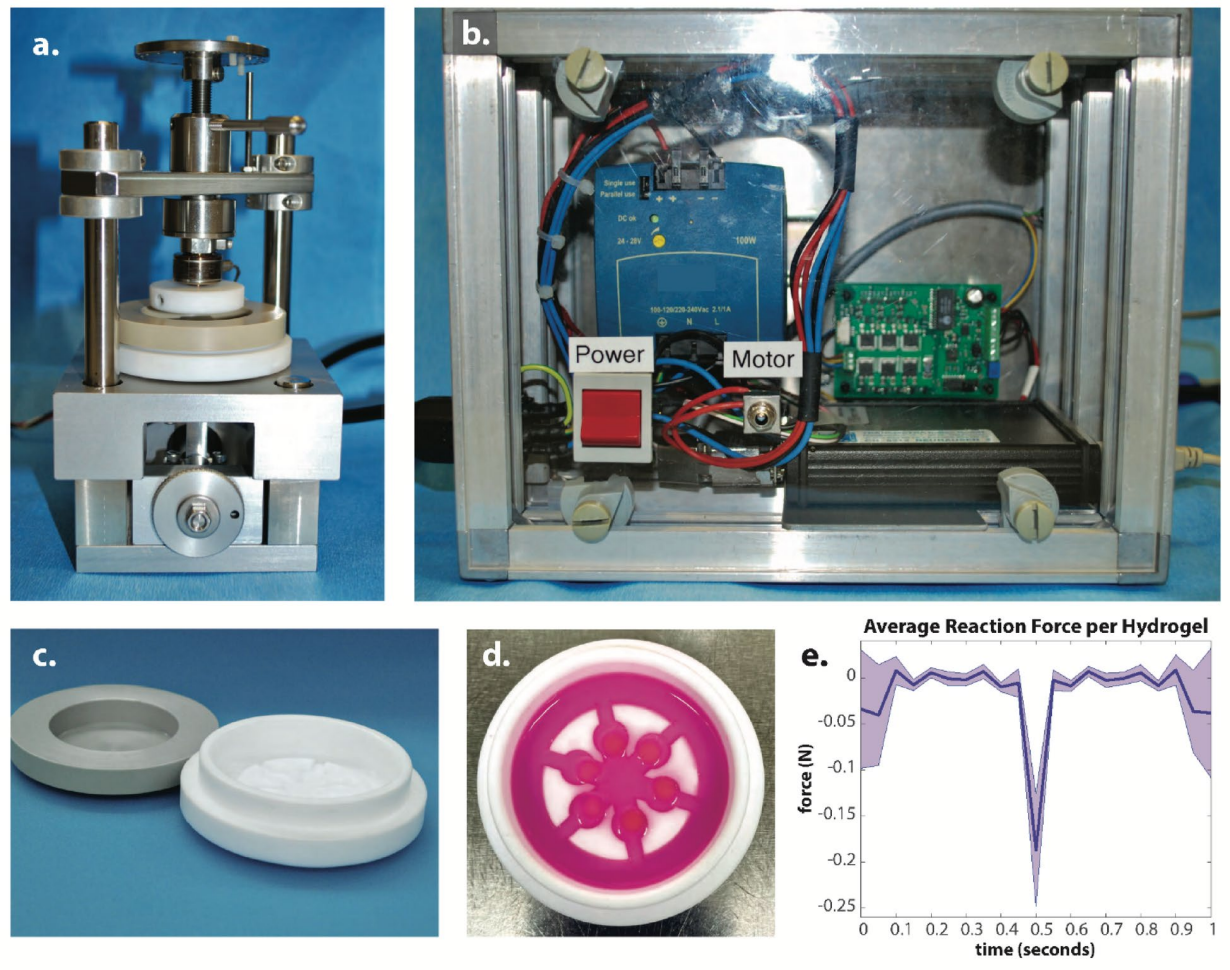
Experimental setup with bioreactor. (**a**) Strain controlled bioreactor with chamber and force cell. (**b**) Power supply and controls of the bioreactor. (**c**) Custom-made chamber for culture of hydrogels and dynamic compression in the bioreactor. (**d**) Custom-made chamber with 6 agarose hydrogels and media solution. (**e**) Average reaction force output for each single hydrogel during one cycle of dynamic compression.

After the phenotype recovery period, the cell-agarose hydrogels were transferred to custom-made unconfined chambers (Fig. 6c, d) and cultured in serum-free LG-DMEM supplemented with 1X ITS+ (1% Insulin, Transferrin, Selenium, BSA and linoleic acid, I2521, Sigma), 1X non-essential amino acids (11140-035, Gibco), 50 ug/mL (172 µM) L-ascorbic acid 2-phosphate (A8960, Sigma), and 10^− 7^ M dexamethasone (D4902, Sigma)^76^ TNF-stimulated hydrogels were treated with 10 ng/mL of TNF (#300–01 A, Peprotech) throughout the duration of the experiment. A custom-made bioreactor (Berner Fachochscule, Burgdorf, Switzerland)^77^was used to dynamically compress the hydrogels at 7% strain at 1.5 Hz for one hour five days per week for up to two weeks (Fig. 6a, b). 7% strain was chosen as it is within the ≤ 20% anabolic physiological range for cell strain in cartilage^63,64^ and did not produce cracks or breakage within the hydrogel during initial loading tests. Each loaded chamber underwent a reaction force of ~ 1.2 N at each compression cycle, thus each loaded hydrogel experienced a force of ~ 0.2 N at each compression cycle (Fig. 6e). Unloaded hydrogels experienced only the constant weight of the chamber lid exerting ~ 5.1 Pa per hydrogel. After 7 or 14 days of culture, hydrogels of each condition were collected 24 h after the last loading cycle for downstream analysis of cell viability, GAG and DNA quantification, and qPCR. Timepoints of 7 and 14 days were selected to allow for changes to be seen biochemically as well as at the gene level^62,78^.

### Cell viability

To determine cell viability of CEP cells in cell-agarose hydrogels on Days 0, 7, and 14, the hydrogels were cultured in serum-free LG-DMEM containing 5 µM calcein-AM (#17783-1MG; Sigma-Aldrich) to stain living cells and 1 µM ethidium homodimer (#46043-1MG-F; Sigma-Aldrich) to stain dead cells and incubated at 37 °C for 35 min. After incubation and washing carriers with PBS, 3D stacked images were taken on a confocal laser scanning microscope (cLSM710; Carl Zeiss; Jena, Germany) at the center and edge of the hydrogel. The maximum number of live and dead cells was determined in each slice-stack measurement and slices between the first and last slices with over half of the maximum total cell number were examined using a custom-made macro for Fiji^79^.

### Cell metabolic activity

To evaluate metabolic activity of CEP cells in cell-agarose hydrogels on Days 0, 7, and 14, the hydrogels were cultured in LG-DMEM supplemented 10% FCS containing 50 µM resazurin sodium salt solution and incubated for three hours at 37 °C. The optical fluorescence index was measured at 544 nm excitation wavelength and 578 nm emission wavelength using an ELISA plate reader (Spectramax M5, Molecular Devices, distributed by Bucher Biotec, Basel, Switzerland). The fluorescent readout was normalized to DNA content before comparison between groups. As soon as the relative fluorescence (RFU) was measured, the hydrogels were washed and immersed in 0.625 mL papain solution per hydrogel, which was composed of 3.9 U/mL papain (Sigma- Aldrich; #P-3125) and 5 mM L-cysteine hydrochloride (Sigma-Aldrich; #1161509). The hydrogels were then digested over night at 60 °C and stored at −20 °C until used to determine the DNA and GAG content.

### GAG and DNA quantification

Hydrogels were collected on days 0, 7, and 14 for GAG and DNA quantification while media was collected during media changes throughout the duration of the experiment and analyzed for GAG quantification of the first media change and the media on days 7 and 14. Media was fully changed every 2–3 days. The amount of GAG produced in the hydrogels (after digestion in papain as described above) and the amount of GAG released into the media was determined using 1,9-dimethyl- methylene blue (Sigma-Aldrich; #341088). Absorbance was measured at a wavelength of 600 nm and results were interpolated using a standard curve based on chondroitin sulphate (Sigma-Aldrich; #C6737).

The DNA content was measured from digested hydrogels using Hoechst 33,258 dye (#86d1405; Sigma-Aldrich). The optical fluorescence index was measured at 350 nm excitation and 450 nm emission wavelengths in digested samples and results were interpolated using a standard curve based on increasing DNA sodium salt concentrations from calf thymus (#D1501; Sigma-Aldrich).

### RNA extraction and qPCR

On days 0, 7, and 14 cell-agarose hydrogels were snap-frozen in liquid nitrogen for downstream analysis of gene expression. Hydrogels were pulverized using a precooled mortar and transferred into 1 ml of Trizol (Life Technologies) for RNA extraction using the GenEluteTM Mammalian Total RNA Miniprep kit (Sigma-Aldrich; #RTN70-1KT). Genomic DNA was digested using on column DNase I (Sigma-Aldrich; #DNASE70-1SET). RNA was then retro-transcribed to complimentary DNA (cDNA) using the High-Capacity cDNA kit (Thermo Fisher Scientific; #4368814) with a MyCyclerTM Thermal Cycler system (Bio-Rad; #1709703). For subsequent qPCR, the cDNA was mixed with the selected relevant primers (see Supplementary Table S2) as well as iTaq Universal SYBR Green Supermix (Bio-Rad; #1725122). Finally, the qPCR was performed using a CFX96TM Real-Time System (Bio-Rad; #185–5096) and the relative gene expression was determined with the 2^−ΔΔCt^ method and normalized to the 18 S reference gene^80^ and the corresponding day 0 value.

### Luminex secretome analysis

Conditioned media was collected during media changes of four different donors throughout the duration of the experiment for each condition and analyzed for 73 secreted proteins using bead-based Luminex multiplex immune-assays separated into six separate secretome panels (see Supplementary Table S3). Assays were developed by Protavio as previously described^81^ For each panel, a 96 well plate was coated with 50 µl of 1X capture antibody bead mix (MagPlex^®^, Luminex Corp, Austin, TX, USA) and then incubated with 35 µl of each undiluted sample, standard, or positive and negative controls at RT for 90 min on an orbital shaker at 1000 rpm. After incubation, wells were washed twice with assay buffer (PR-ASSB-1x, Protavio, Greece) to remove unbound material. 20 µl of detection antibody mix was then added followed by incubation at RT for 60 min on an orbital shaker at 1000 rpm. After incubation, wells were washed twice with assay buffer, and 35 µl of streptavidin-phycoerythrin conjugate (SAPE, S866, Invitrogen, USA) at a 1:200 dilution was added, followed by 15 min of incubation at room temperature (RT) on an orbital shaker at 1000 rpm. Finally, wells were washed twice with assay buffer, resuspended in 130 µl assay buffer, and measured in a FlexMAP 3D instrument (Luminex Corp, Austin, TX, USA) using a minimum of 100 counts. Median fluorescence intensity (MFI) was used for subsequent statistical analysis. For absolute quantification of cytokine levels in the secretome, net MFI values were calculated by subtracting MFI values of the blanks for each marker and then standards were used to generate calibration curves through a 5PL logistic regression.

### Luminex phosphoprotein analysis

Hydrogels were collected on days 0, 7, and 14 for each condition for two different donors and protein lysates were extracted following the protocol by Bougault et al.,^68^ using a lysis buffer containing protease and phosphatase inhibitors (Protavio) in place of Laemmli buffer. A Pierce bicinchoninic acid assay (BCA) protein assay (A65453, Thermo Fisher Scientific) was used to quantify protein content. Samples were diluted to a protein concentration of 300 µg/ml and analyzed for 21 phosphorylated proteins using bead-based Luminex multiplex immune-assays separated into two separate phospho-panels for semi-quantitative measurement of phosphorylated proteins (see Supplementary Table S4). Panels were executed using the protocol in the previous section (see Luminex Secretome Analysis). Positive (signal) and negative (noise) control lysates were used to assess the capture and detection antibodies in each assay. MFI values of a blank (lysates from an agarose hydrogel with no cells) were subtracted from corresponding MFI values for each marker (see Supplementary Figure S1).

### Regulatory network model

Additionally, a previously developed AC chondrocyte mechanotransduction RNM^28^ was used to predict protein activation levels for comparison to matched experiment secretome data (Supplementary Table S1). Protein activation levels were predicted based on sustained perturbations (set between 0 and 1, with 0 being full knock-down, and 1 being full activation) of the nodes that represent the proteins involved in the imposed experimental conditions, including TNF stimulation and compression. To replicate the hydrogels at day 0, random initial conditions were assumed for all nodes, except for those that aim to simulate the assumed basal cell-matrix interactions (Table 2), and the system was solved 100 times to find its attractors, i.e., representative steady states. Similarly, simulations of the experimental perturbations (i.e. control unloaded, control dynamic, TNF unloaded, TNF dynamic) were performed (Table 2). The responses for each condition were evaluated to estimate the percentage of steady states that were pro-anabolic (e.g., ACAN ≥ 0.5) or pro-catabolic (e.g., ACAN < 0.5).

**Table 2:** Setup for literature-derived and experiment-derived RNM simulations.

Treatment	Mechanical Loading	Clamped nodes
Literature-derived		
Day 0		α5β1 = 0.5 αvβ3 = 0.5 TRPV4 = 0.5
Control	Unloaded	α5β1 = 0.5 αvβ3 = 0.5 TRPV4 = 0.5
	Dynamic	α5β1 = 1 αvβ3 = 1 TRPV4 = 1
TNF	Unloaded	α5β1 = 0.5 αvβ3 = 0.5 TRPV4 = 0.5 TNF = 1
	Dynamic	α5β1 = 1 αvβ3 = 1 TRPV4 = 1 TNF = 1
Experiment-derived		
Day 0		α5β1 = 0.5 TRPV4 = 1 Actin = 0 IL4 = 0.2585
Control	Unloaded	α5β1 = 0.5 TRPV4 = 0.5 Actin = 0 IL4 = 0.2585
	Dynamic	(same as unloaded)
TNF	Unloaded	α5β1 = 0.5 TRPV4 = 0.25 Actin = 0 IL4 = 0.2585 TNF = 1
	Dynamic	(same as unloaded)

Two different approaches (Table 2) were taken to produce the initial node setup for the RNM simulations: (1) activated mechanoreceptors were derived from literature on AC chondrocytes in agarose; and (2) activated mechanoreceptors were determined based on gene expression results of TRPV4 and integrin subunits α5 and β1 as well as low IL-4 detected in the secretome and lack of actin to represent deficient mechanotransduction. Low IL-4 was established as the critical expression level at which IL-4 is able to change from a pro-catabolic to a pro-anabolic steady state, or vice versa (i.e., ≈ 0.2585). For the literature-based approach, mechanoreceptors were chosen to represent free swelling for the baseline (TRPV4 and integrin αvβ3)^34,71^ dynamic compression (integrins α5β1, αvβ3),^71^ and physio-osmotic pressure (TRPV4)^34^ Integrin αVβ5^84^ and PIEZO1/2^85,86^ were not selected because the strain was not determined to be excessive^64^.

### Semi-quantitative comparison of RNM and experiment results

Protein activation levels from the RNM simulations for both the literature-derived and experiment-derived approaches were semi-quantitatively compared to protein secretion values using normalized mean absolute deviation (NMAD) to measure the cumulative error between the predicted and the experimental datasets, and the normalized root squared error (NRSE) following methods established by Segarra-Queralt et al.^36^ In brief, experiment secretome results were normalized calibrated between 0 and 1, representing the lower and upper limits of quantification of each protein in the Luminex assay; while RNM results were averaged among the 100 iterations for each experimental condition. For both experimental and RNM outputs, the control unloaded condition was considered as a reference value and subtracted from all other conditions, positioning the data range between − 1 and 1. NMAD was calculated using Eq. 1 while NRSE was calculated using Eq. 2.

1 $$NMAD = \frac{{\sum\nolimits_{n}^{N} {\left| {x_{n} - y_{n} } \right|} }}{N}$$

2 $$NRSE_{n} = \frac{{\sqrt {\left( {x_{n} + y_{n} } \right)^{2} } }}{{MAX.RSE}}$$

### Quantification and statistical analysis

All statistical analyses for gene expression, secretome quantification, GAG quantification, cell metabolism, and cell viability were performed assuming a non-parametric distribution. Thus, a Kruskal-Wallis test followed by a Dunn’s multiple comparisons *post-hoc* test was used to evaluate results. A Benjamini-Hochberg correction was used to correct for multiple comparisons within the factors of treatment, loading, and time. Gene expression, GAG, and cell metabolic data were normalized to the respective Day 0 and Day 7 unloaded control condition, and finally log-transformed prior to statistical analysis. Secretome data was normalized to the Day 7 control unloaded condition and log-transformed prior to statistical analysis. All statistical analyses were performed using R (R Core Team, 2020) and RStudio (R Version 4.4.0, R Studio Team, 2020), specifically the R stats and “dunn.test” packages^85^ A *p*-value < 0.05 was considered statistically significant. All quantitative results are presented as median up to six biological replicates and exact number of biological replicates (n) is indicated in each figure legend.

## Electronic supplementary material

Below is the link to the electronic supplementary material.

Supplementary Material 1

## Data Availability

The regulatory network model used in this manuscript can be found at doi: 10.3389/fbioe.2023.1006066. Any additional information required to reanalyze the data reported in this paper is available from the lead contact upon request.
