# Supplementary material for: TNF induces catabolism in human cartilaginous endplate cells in 3D agarose culture under dynamic compression
Source: Sci Rep. 2025 May 6;15:15849. doi: 10.1038/s41598-025-00538-w (PMC12056083; doi:10.1038/s41598-025-00538-w)

Table S1. Regulatory network outputs
Proteins in bold and italic were used for comparison to the experiment gene expression and protein secretion data. Related to Figure 5.

	Literature-Derived					Experiment-Derived		
**Node**	**Baseline D0 SS**	**Control Static SS**	**Control Dynamic SS**	**TNF Static SS**	**TNF Dynamic SS**	**Baseline D0 SS**	**Control SS**	**TNF SS**
a10b1	8.201E-01	8.201E-01	9.101E-01	2.661E-02	4.396E-02	6.548E-01	6.547E-01	6.413E-03
a1b1	8.458E-01	8.458E-01	1.000E+00	8.458E-01	1.000E+00	1.000E+00	8.458E-01	2.658E-01
a2b1	6.420E-65	6.420E-65	6.420E-65	6.420E-65	6.420E-65	6.420E-65	6.420E-65	6.420E-65
a5b1	5.000E-01	5.000E-01	1.000E+00	5.000E-01	1.000E+00	5.000E-01	5.000E-01	5.000E-01
AC	1.245E-03	1.245E-03	7.327E-04	1.140E-03	6.760E-04	6.019E-04	1.024E-03	1.157E-03
ACAN	***8.200E-01***	***8.200E-01***	***9.100E-01***	***2.385E-02***	***3.945E-02***	***4.918E-01***	***4.918E-01***	***8.257E-03***
Actin	8.073E-01	8.073E-01	9.108E-01	8.423E-02	1.922E-01	0.000E+00	0.000E+00	0.000E+00
ADAMT4	1.796E-01	1.796E-01	8.978E-02	8.726E-01	7.998E-01	5.481E-01	5.481E-01	9.899E-01
ADAMT5	***1.791E-01***	***1.791E-01***	***8.954E-02***	***8.716E-01***	***7.798E-01***	***5.289E-01***	***5.289E-01***	***9.790E-01***
AK	9.720E-01	9.720E-01	9.997E-01	9.060E-01	9.989E-01	9.885E-01	9.885E-01	9.885E-01
Akt	1.775E-01	1.775E-01	8.877E-02	9.171E-01	8.727E-01	5.113E-01	5.114E-01	9.774E-01
AP1	1.797E-01	1.797E-01	8.987E-02	9.953E-01	9.927E-01	5.587E-01	5.587E-01	9.979E-01
ATP	5.026E-64	5.026E-64	5.026E-64	5.026E-64	5.026E-64	5.026E-64	5.026E-64	5.026E-64
aVb3	5.000E-01	5.000E-01	1.000E+00	5.000E-01	1.000E+00	4.024E-01	4.024E-01	2.467E-05
aVb5	1.800E-01	1.800E-01	9.000E-02	6.100E-01	3.601E-01	1.449E-01	1.452E-01	7.728E-01
Bcatenin	2.967E-63	2.967E-63	2.951E-63	2.130E-63	1.585E-63	1.739E-63	1.742E-63	2.797E-63
BMP2	1.744E-01	1.744E-01	8.723E-02	5.940E-01	3.512E-01	1.259E-01	1.260E-01	3.893E-01
BMP2R	1.798E-01	1.798E-01	8.990E-02	6.097E-01	3.599E-01	1.150E-01	1.153E-01	6.486E-01
Ca	9.616E-01	9.616E-01	9.790E-01	9.523E-01	9.790E-01	9.789E-01	9.654E-01	9.541E-01
Calmodulin	9.985E-01	9.985E-01	9.992E-01	9.981E-01	9.992E-01	9.992E-01	9.987E-01	9.982E-01
CAMK	9.335E-01	9.335E-01	9.317E-01	9.428E-01	9.376E-01	9.306E-01	9.305E-01	9.314E-01
cAMP	1.696E-04	1.696E-04	9.940E-05	1.552E-04	9.166E-05	8.156E-05	1.394E-04	1.575E-04
CASP8	1.522E-01	1.522E-01	7.610E-02	5.159E-01	3.045E-01	1.150E-02	1.150E-02	2.080E-01
CD40	6.224E-65	6.224E-65	6.224E-65	6.224E-65	6.224E-65	6.224E-65	6.224E-65	6.224E-65
CITED2	8.618E-01	8.618E-01	9.292E-01	5.353E-01	7.202E-01	8.431E-01	8.430E-01	6.612E-01
COL2A	***8.208E-01***	***8.208E-01***	***9.104E-01***	***5.735E-02***	***9.196E-02***	***6.036E-01***	***6.036E-01***	***3.476E-02***
COMP	6.482E-65	6.482E-65	6.482E-65	6.482E-65	6.482E-65	6.482E-65	6.482E-65	6.482E-65
ConHem43	6.764E-65	6.764E-65	6.764E-65	6.764E-65	6.764E-65	6.764E-65	6.764E-65	6.764E-65
CREB	9.996E-01	9.996E-01	9.996E-01	9.997E-01	9.996E-01	9.996E-01	9.996E-01	9.996E-01
CYCS	1.800E-01	1.800E-01	8.999E-02	6.100E-01	3.600E-01	2.761E-01	2.761E-01	5.266E-01
DAG	9.249E-01	9.249E-01	9.989E-01	7.454E-01	9.956E-01	9.975E-01	9.975E-01	9.921E-01
DDR2	1.800E-01	1.800E-01	9.000E-02	9.944E-01	9.907E-01	5.429E-01	5.430E-01	9.988E-01
Dishwelsed	1.652E-63	1.652E-63	1.652E-63	1.652E-63	1.652E-63	1.652E-63	1.652E-63	1.652E-63
Endoglin	1.800E-01	1.800E-01	9.000E-02	9.979E-01	9.961E-01	5.599E-01	5.599E-01	1.000E+00
ERK	9.995E-01	9.995E-01	9.998E-01	9.994E-01	9.998E-01	9.998E-01	9.996E-01	9.995E-01
FAK	1.406E-01	1.406E-01	7.029E-02	4.764E-01	2.812E-01	4.213E-02	4.230E-02	6.119E-01
Fibronectin	8.199E-01	8.199E-01	9.099E-01	1.100E-01	1.702E-01	2.532E-01	2.532E-01	1.828E-04
FOXO	8.195E-01	8.195E-01	9.100E-01	1.571E-03	3.859E-03	6.392E-65	6.392E-65	6.392E-65
Frizzled	4.249E-64	4.249E-64	4.249E-64	4.249E-64	4.249E-64	4.249E-64	4.249E-64	4.249E-64
FrzB	6.403E-65	6.403E-65	6.403E-65	6.403E-65	6.403E-65	6.403E-65	6.403E-65	6.403E-65
FZD6	1.790E-02	1.790E-02	8.947E-03	6.069E-02	3.585E-02	3.511E-03	3.511E-03	7.325E-03
GLIa	2.924E-63	2.924E-63	3.257E-63	1.425E-63	2.214E-63	5.348E-64	5.449E-64	9.932E-65
GLIr	6.297E-63	6.297E-63	6.817E-63	4.015E-63	5.215E-63	2.369E-63	2.379E-63	1.943E-63
GPCR	9.004E-01	9.004E-01	9.936E-01	6.901E-01	9.746E-01	8.173E-01	8.173E-01	8.173E-01
Grb2	1.761E-01	1.761E-01	8.804E-02	9.239E-01	8.891E-01	4.841E-01	4.842E-01	9.681E-01
GsK3b	1.000E+00	1.000E+00	1.000E+00	1.000E+00	1.000E+00	1.000E+00	1.000E+00	1.000E+00
HDAC4	7.625E-01	7.625E-01	8.462E-01	3.626E-01	5.951E-01	4.092E-01	4.092E-01	3.616E-06
HIF1	1.000E+00	1.000E+00	1.000E+00	1.000E+00	1.000E+00	1.000E+00	1.000E+00	1.000E+00

HIF2a	1.789E-01	1.789E-01	8.947E-02	6.097E-01	3.600E-01	6.217E-02	6.228E-02	3.011E-01
IFNg	***1.789E-01***	***1.789E-01***	***8.947E-02***	***6.097E-01***	***3.600E-01***	***6.217E-02***	***6.228E-02***	***3.011E-01***
IGF1	8.200E-01	8.200E-01	9.100E-01	3.862E-01	6.377E-01	4.411E-01	4.411E-01	9.279E-05
Ihh	1.748E-01	1.748E-01	8.742E-02	5.928E-01	3.500E-01	4.538E-01	4.539E-01	8.859E-01
Ikbkin	1.757E-01	1.757E-01	8.814E-02	8.573E-01	6.034E-01	4.938E-01	4.938E-01	9.796E-01
IL10	***8.198E-01***	***8.198E-01***	***9.098E-01***	***3.895E-01***	***6.393E-01***	***2.879E-01***	***2.879E-01***	***2.879E-01***
IL13	***8.198E-01***	***8.198E-01***	***9.098E-01***	***3.895E-01***	***6.393E-01***	***2.879E-01***	***2.879E-01***	***2.879E-01***
IL13R	8.198E-01	8.198E-01	9.098E-01	3.895E-01	6.393E-01	2.879E-01	2.879E-01	2.879E-01
IL17	***1.800E-01***	***1.800E-01***	***9.000E-02***	***9.998E-01***	***9.997E-01***	***5.600E-01***	***5.600E-01***	***9.999E-01***
IL18	***1.789E-01***	***1.789E-01***	***8.944E-02***	***6.062E-01***	***3.578E-01***	***2.241E-01***	***2.241E-01***	***4.281E-01***
IL1b	***6.193E-02***	***6.193E-02***	***3.096E-02***	***2.130E-01***	***1.289E-01***	***9.116E-02***	***9.117E-02***	***1.728E-01***
IL1bR	4.231E-02	4.231E-02	2.115E-02	1.437E-01	8.516E-02	2.580E-02	2.581E-02	5.186E-02
IL1Ra	8.199E-01	8.199E-01	9.099E-01	3.846E-01	6.368E-01	4.603E-01	4.602E-01	3.300E-02
IL4	***8.138E-01***	***8.138E-01***	***9.047E-01***	***3.752E-01***	***6.218E-01***	***2.585E-01***	***2.585E-01***	***2.585E-01***
IL4R	8.198E-01	8.198E-01	9.098E-01	3.894E-01	6.393E-01	1.817E-01	1.816E-01	2.075E-02
IL6	***1.794E-01***	***1.794E-01***	***8.971E-02***	***6.081E-01***	***3.589E-01***	***9.357E-02***	***9.358E-02***	***1.847E-01***
IL8	***2.768E-02***	***2.768E-02***	***1.384E-02***	***9.879E-02***	***6.352E-02***	***1.234E-01***	***1.235E-01***	***2.907E-01***
IP3	9.249E-01	9.249E-01	9.989E-01	7.454E-01	9.956E-01	9.975E-01	9.975E-01	9.921E-01
JAK	8.200E-01	8.200E-01	9.100E-01	3.900E-01	6.400E-01	4.421E-01	4.419E-01	1.839E-01
JNK	9.338E-01	9.338E-01	9.318E-01	9.432E-01	9.377E-01	9.308E-01	9.308E-01	9.318E-01
LIF	***1.800E-01***	***1.800E-01***	***9.000E-02***	***1.000E+00***	***1.000E+00***	***1.311E-01***	***1.312E-01***	***1.000E+00***
Mek	9.999E-01	9.999E-01	9.999E-01	1.000E+00	1.000E+00	1.000E+00	1.000E+00	1.000E+00
MMP1	***1.676E-01***	***1.676E-01***	***8.380E-02***	***5.691E-01***	***3.370E-01***	***3.429E-02***	***3.430E-02***	***7.925E-02***
MMP13	1.635E-01	1.635E-01	8.175E-02	5.542E-01	3.271E-01	3.018E-02	3.020E-02	6.995E-02
MMP14	1.789E-01	1.789E-01	8.943E-02	6.075E-01	3.600E-01	9.838E-02	9.838E-02	1.830E-01
MMP3	***1.622E-01***	***1.622E-01***	***8.108E-02***	***5.531E-01***	***3.300E-01***	***1.033E-01***	***1.034E-01***	***3.097E-01***
NFkB	1.583E-01	1.583E-01	7.927E-02	5.533E-01	3.304E-01	9.771E-02	9.779E-02	2.635E-01
NO	1.793E-01	1.793E-01	8.964E-02	6.081E-01	3.592E-01	2.795E-01	2.795E-01	6.027E-01
P2R	2.300E-63	2.300E-63	2.300E-63	2.300E-63	2.300E-63	2.300E-63	2.300E-63	2.300E-63
p38	1.000E+00	1.000E+00	1.000E+00	1.000E+00	1.000E+00	1.000E+00	1.000E+00	1.000E+00
PC2	8.592E-63	8.592E-63	8.592E-63	8.592E-63	8.592E-63	8.592E-63	8.592E-63	8.592E-63
PGA	1.239E-05	1.239E-05	6.178E-06	4.959E-04	7.558E-04	5.784E-03	5.784E-03	7.197E-03
PGE2	1.784E-01	1.784E-01	8.920E-02	6.049E-01	3.572E-01	2.085E-01	2.085E-01	4.000E-01
PI3K	1.794E-01	1.794E-01	8.971E-02	9.697E-01	9.523E-01	5.296E-01	5.296E-01	9.926E-01
PIEZO	5.948E-65	5.948E-65	5.948E-65	5.948E-65	5.948E-65	5.948E-65	5.948E-65	5.948E-65
PKA	2.113E-05	2.113E-05	1.295E-05	1.480E-05	1.040E-05	9.563E-06	1.636E-05	1.498E-05
PKC	9.956E-01	9.956E-01	9.995E-01	9.958E-01	9.996E-01	9.964E-01	9.962E-01	9.994E-01
PLC	8.853E-01	8.853E-01	9.829E-01	6.115E-01	9.317E-01	9.423E-01	9.422E-01	8.553E-01
PPR	8.200E-01	8.200E-01	9.100E-01	3.899E-01	6.399E-01	4.404E-01	4.404E-01	2.079E-05
PTCH	1.865E-63	1.865E-63	2.061E-63	9.606E-64	1.424E-63	4.860E-64	5.025E-64	1.859E-64
PTHrP	8.200E-01	8.200E-01	9.100E-01	3.869E-01	6.384E-01	4.423E-01	4.422E-01	1.540E-04
Rack	5.855E-01	5.855E-01	9.359E-01	7.896E-01	9.540E-01	5.665E-01	5.667E-01	9.538E-01
Raf	9.975E-01	9.975E-01	9.973E-01	1.000E+00	1.000E+00	9.987E-01	9.987E-01	1.000E+00
Ras	9.415E-01	9.415E-01	9.361E-01	9.999E-01	9.999E-01	9.686E-01	9.686E-01	1.000E+00
RGD	1.798E-01	1.798E-01	8.989E-02	6.097E-01	3.603E-01	1.384E-01	1.385E-01	4.496E-01
Rho	1.771E-01	1.771E-01	8.856E-02	6.002E-01	3.542E-01	1.132E-02	1.139E-02	9.362E-01
ROCK	8.199E-01	8.199E-01	9.101E-01	5.526E-02	2.501E-01	6.198E-65	6.198E-65	6.198E-65
ROS	1.624E-01	1.624E-01	8.122E-02	8.536E-01	8.191E-01	1.059E-01	1.060E-01	8.563E-01
Runx2	1.783E-01	1.783E-01	8.935E-02	6.054E-01	3.588E-01	5.320E-01	5.321E-01	9.713E-01
SAC	6.340E-65	6.340E-65	6.340E-65	6.340E-65	6.340E-65	6.340E-65	6.340E-65	6.340E-65
Smad	4.351E-64	4.351E-64	4.351E-64	4.351E-64	4.351E-64	4.351E-64	4.351E-64	4.351E-64
Smad15	1.749E-01	1.749E-01	8.746E-02	5.928E-01	3.499E-01	1.448E-01	1.454E-01	9.175E-01
Smad23	8.200E-01	8.200E-01	9.100E-01	1.862E-07	3.875E-07	4.400E-01	4.400E-01	4.723E-16
SMO	1.798E-01	1.798E-01	8.991E-02	6.094E-01	3.597E-01	5.531E-01	5.531E-01	9.944E-01
Sos	1.799E-01	1.799E-01	8.993E-02	9.959E-01	9.937E-01	5.560E-01	5.560E-01	9.988E-01
Sox9	***8.200E-01***	***8.200E-01***	***9.100E-01***	***3.329E-01***	***6.007E-01***	***4.429E-01***	***4.428E-01***	***1.132E-03***

SP	6.986E-01	6.986E-01	9.163E-01	3.469E-01	6.652E-01	5.978E-01	5.973E-01	5.749E-02
STAT6	8.193E-01	8.193E-01	9.101E-01	3.868E-01	6.383E-01	5.423E-01	5.418E-01	1.767E-02
SUFU	4.312E-63	4.312E-63	4.759E-63	2.308E-63	3.362E-63	1.032E-63	1.029E-63	5.610E-64
TBRI	7.690E-01	7.690E-01	8.494E-01	3.869E-01	6.124E-01	7.162E-01	7.157E-01	7.720E-02
TBRII	8.200E-01	8.200E-01	9.100E-01	4.124E-06	8.574E-06	4.400E-01	4.400E-01	2.368E-14
TGFb	***8.200E-01***	***8.200E-01***	***9.100E-01***	***1.177E-02***	***2.144E-02***	***4.407E-01***	***4.407E-01***	***1.615E-06***
TIMP	8.089E-01	8.089E-01	8.977E-01	5.270E-03	8.746E-03	4.135E-01	4.135E-01	2.477E-03
TLR4	1.800E-01	1.800E-01	9.000E-02	9.944E-01	9.907E-01	5.429E-01	5.430E-01	9.988E-01
TNF	1.799E-01	1.799E-01	8.997E-02	1.000E+00	1.000E+00	1.328E-01	1.329E-01	1.000E+00
TRPV4	5.000E-01	5.000E-01	1.000E+00	5.000E-01	1.000E+00	1.000E+00	5.000E-01	2.500E-01
Tyrkin	6.013E-65	6.013E-65	6.013E-65	6.013E-65	6.013E-65	6.013E-65	6.013E-65	6.013E-65
VEGF	***1.794E-01***	***1.794E-01***	***8.971E-02***	***6.080E-01***	***3.588E-01***	***1.317E-01***	***1.318E-01***	***3.158E-01***
Wnt	6.712E-65	6.712E-65	6.712E-65	6.712E-65	6.712E-65	6.712E-65	6.712E-65	6.712E-65
Wnt5a	6.490E-65	6.490E-65	6.490E-65	6.490E-65	6.490E-65	6.490E-65	6.490E-65	6.490E-65

Table S2. qPCR primer sequences

Gene Type	Gene	Gene ID	Forward and Reverse
Reference gene	18S	*18S*	f- CGA TGC GGC GGC GTT ATT C<br>r- TCT GTC AAT CCT GTC CGT GTC C
Anabolic Markers	Aggrecan	*ACAN*	f- CAT CAC TGC AGC TGT CAC<br>r- AGC AGC ACT ACC TCC TTC
	Type I collagen	*COL1A2*	f- GTG GCA GTG ATG GAA GTG<br>r- CAC CAG TAA GGC CGT TTG
	Type II collagen	*COL2A1*	f- AGC AGC AAG AGC AAG GAG AA<br>r- GTA GGA AGG TCA TCT GGA
	Type VI collagen	*COL6A1*	f- TTCAAGGAGGCTGTCAAGAAC<br>r- TGATGAGGCGGTCGTAGG
	SRY-Box Transcription Factor 9	*SOX9*	f- GAG ACT TCT GAA CGA GAG<br>r- GCT CTG ATG TGT TGA AGA AC
Catabolic Markers	A disintegrin and metalloproteinase with thrombospondin motifs 5	*ADAMTS5*	f- GCT GTG CTG TGA TTG AAG A<br>r- TGC TGG TAA GGA TGG AAG A
	Matrix metalloproteinase-3	*MMP-3*	f- CAA GGC ATA GAG ACA ACA TAG A<br>r- GCA CAG CAA CAG TAG GAT
	Interleukin 6	*IL6*	f- GCC ACT CAC CTC TTC AGA AC<br>r- GCA AGT CTC CTC ATT GAA TCC A
	Type X Collagen	*COLX*	f- GAA TGC CTG TGT CTG CTT<br>r- TCA TAA TGC TGT TGC CTG TTA
Mechanoreceptors	Transient receptor potential cation channel subfamily V member 4	*TRPV4*	f- GTT GGT CTG GTC CTC ATT G<br>r- GAT TCC TGC TCG TCT ACT TG
	Integrin Subunit Alpha 5	*ITGA5*	f- ATC GCT CTC AAC TTC TCC TT<br>r- CGG CTC TTG CTC TGA TAA TG
	Integrin Subunit Beta 1	*ITGB1*	f- CCT TGG TGT CTG TGC TGA<br>r- GTC GTC AAC ATC CTT CTC CTT AC

Table S3. Performance characteristics of Luminex multiplex cyto-plex panels
Abbreviations: LOD, Limit of detection; LLOQ, Low limit of quantification; ULOQ, Upper limit of quantification.

	Gene name	Cytokine	Uniprot ID	Protein Name	LOD (pg/mL)	LLOQ (pg/mL)	ULOQ (pg/mL)
PANEL 1	IL4	IL4	P05112	Interleukin-4	23.1	23.1	5500
	IL1B	IL1b	P01584	Interleukin-1 beta	1	3	1900
	IL1A	IL1A	P01583	Interleukin-1 alpha	2	3	1900
	IFNG	IFNG	P01579	Interferon gamma	10.2	10.2	5500
	CSF3R	G-CSF	P09919	Granulocyte colony-stimulating factor	32.3	32.3	5500
	SCF	SCF	P21583	Kit ligand	0.7	0.7	600
	IL17F	IL17F	Q96PD4	Interleukin-17F	21.1	23	16600
	IL7	IL7	P13232	Interleukin-7	1.7	3	5500
	IL16	IL16	Q14005	Interleukin-16	1.9	3	5500
	IL18	IL18	Q14116	Interleukin-18	0.5	1	1900
	IL6	IL6	P05231	Interleukin-6	0.4	1	1900
	PROK1	PROK1	Q9HC23	Prokineticin-1	3.6	8	16600
	TNFA	TNFA	P01375	Tumor necrosis factor	1.5	3	5500
	IL20	IL20	Q9NYY1	Interleukin-20	1.9	8	16600
	FGF2	FGF BASIC	P09038	Fibroblast growth factor 1	6.3	8	5500
	IL10	IL10	P22301	Interleukin-10	0.1	3	5500
	IL1RA	IL1RA	P18510	Interleukin-1 receptor antagonist protein	23	23	5500
PANEL 2	CCL19	CCL19	P78556	C-C motif chemokine 19	39.6	99	8000
	CXCL13	CXCL13	O43927	C-X-C motif chemokine 13	69.7	69.7	16600
	CXCL12	CXCL12	P48061	Stromal cell-derived factor 1	193.2	206	50000
	CSF2	GM-CSF	P04141	Granulocyte-macrophage colony-stimulating factor	0.4	1	3200
	CSF1R	M-CSF	P09603	Macrophage colony-stimulating factor 1	0.8	1	3200
	IL13	IL13	P35225	Interleukin-13	10.5	10.5	8000
	IL2RA	IL2RA	P01589	Interleukin-2 receptor subunit alpha	56.9	56.9	50000
	IL15	IL15	P40933	Interleukin-15	0.1	1	1300
	LIF	LIF	P15018	Leukemia inhibitory factor	8.4	8.4	5500
	IFNA2	IFNA2	P01563	Interferon alpha-2	0.5	1	3200
	IL12	IL12	P29459	Interleukin-12 subunit alpha	4.3	4.3	5500
	IL11	IL11	P20809	Interleukin-11	122	122	20000

	IL22	IL22	Q9GZX6	Interleukin-22	0.6	1	3200
	CCL27	CTACK	Q9Y4X3	C-C motif chemokine 27	13.3	23	5500
	CXCL10	IP10	P02778	C-X-C motif chemokine 10, 10 kDa interferon gamma-induced protein	0.3	1	3200
	IL9	IL9	P15248	Interleukin-9	2.8	3	5500
	IL17A	IL17A	Q16552	Interleukin-17A	5.5	5.5	5500
	CCL2	CCL2	P13500	C-C motif chemokine 2, Monocyte chemotactic protein 1	2.5	3	1900
**PANEL 3**	TNFRSF9	TNFRSF9	P41273	Tumor necrosis factor receptor superfamily member 9	2.4	3	5500
	S100A8	S100A8	P05109	Protein S100-A8	8.1	23	50000
	TNF10	TNF10	P50591	Tumor necrosis factor ligand superfamily member 10	7.2	8	16600
	FST	FST	P19883	Follistatin	17.4	68	16600
	NGF	NGF	P01138	Beta-nerve growth factor	19.9	19.9	16600
	MMP13	MMP13	P45452	Collagenase 3	1.8	3	5500
	ST2	ST2	Q9UBE8	Interleukin-1 receptor-like 1	6.5	23	5500
	NRG1	NRG1	Q02297	Pro-neuregulin-1, membrane-bound isoform	3.1	23	50000
	RETN	RETN	Q9HD89	Resistin	4.5	8	16600
	CNTF	CNTF	P26441	Ciliary neurotrophic factor	2.9	8	16600
	MMP1	MMP1	P03956	Interstitial collagenase	7.7	7.7	5500
**PANEL 4**	CCL20	CCL20	P78556	C-C motif chemokine 20	67.6	617	16700
	MIF	MIF	P14174	Macrophage migration inhibitory factor	17.2	23	50000
	CXCL1	GROA	P09341	Growth-regulated alpha protein	0.2	1	1900
	MCP3	CCL7	P80098	C-C motif chemokine 7	36.6	36.6	5500
	CXCL16	CXCL16	O95799	C-X-C motif chemokine 16	6.4	205	5500
	CCL17	CCL17	Q92583	C-C motif chemokine 17	0.32	1	1900
	CCL22	CCL22	O00626	C-C motif chemokine 22	64.5	370	10000

	CCL3	CCL3	P10147	C-C motif chemokine 3, Macrophage inflammatory protein 1-alpha	1.5	1.5	1900
	CXCL11	CXCL11	O14625	C-X-C motif chemokine 11, Interferon-inducible T-cell alpha chemoattractant	8.1	205	16600
	TNF12	TWEAK	O43508	Tumor necrosis factor ligand superfamily member 12	2.2	3	5500
	CXCL9	CXCL9	Q07325	C-X-C motif chemokine 9	0.4	0.4	210
PANEL 5	CCL4	CCL4	P13236	C-C motif chemokine 4	1.8	2	67
	TNFSF11	sRANK-L	O14788	Tumor necrosis factor ligand superfamily member 11	3	3	5500
	VEGF	VEGF	P15692	Vascular endothelial growth factor A	7.5	23	50000
	IL8	IL8	P10145	Interleukin-8	0.9	0.9	620
	IL5	IL5	P05113	Interleukin-5	0.8	1	1850
	MMP7	MMP7	P09237	Matrilysin	3.5	3.5	5500
	TGFB1	TGF-b1	P01137	Transforming growth factor beta-1 protein	1.6	68	16600
	CCL11	Eotaxin	P51671	Eotaxin	1.9	3	5500
PANEL 6	VCAM1	VCAM1	P19320	Vascular cell adhesion protein 1	1.1	1.1	1900
	TIMP1	TIMP1	P01033	Metalloproteinase inhibitor 1	1.1	1.1	1900
	MMP2	MMP2	P08253	72 kDa type IV collagenase	7.6	8	16600
	MMP9	MMP9	P14780	Matrix metalloproteinase-9	3	3	5500
	CCL5	RANTES	P13501	C-C motif chemokine 5	1.6	3	5500
	ICAM1	ICAM1	P05362	Intercellular adhesion molecule 1	0.1	1	1900
	SERPINE1	PAI-1	P05121	Plasminogen activator inhibitor 1	2.6	3	5500
	DEFB1	DEFB1	P60022	Beta-defensin 1	1	3	600

Table S4. Performance assessment of Luminex phospho-plex multiplex panels
Positive and negative control cell lysates were measured by each assay and a 2-fold or greater increase in MFI values in positive lysates compared to negative lysates (SNR) was required for assay qualification.

	Protein	Uniprot ID	Phosphorylated residue	Signal/Noise Ratio (SNR)	Pass/Fail
PANEL 1	SMAD3	P84022	S423/S425	2.83	pass
	P53	P04637	S15	23.91	pass
	CREB1	P16220	S133	45.48	pass
	AKT1S1	Q96B36	T246	162.23	pass
	IKBA	P25963	S32/S36	5.08	pass
	FAK	Q05397	Y397	3.92	pass
	GSK3	P49840	S21	16.65	pass
	AKT1	P31749	S473	18.68	pass
	HSPB1	P04792	S78/S82	531.63	pass
	P38	P78345	T180/Y182	47.75	pass
	mTOR	P42345	S2448	3.36	pass
	MEK1	Q13233	S217/S221	79.79	pass
	RSK1	Q15418	S380	36.02	pass
PANEL 2	JUN	P05412	S63	83.12	pass
	EGFR	P00533	Y1068	109.78	pass
	ERK1	P27361	T202	128.29	pass
	MARCKS	P49006	S170	74.88	pass
	PTN11	Q06124	Y542	85.95	pass
	CHK2	O96017	T68	1.79	pass
	STAT3	P40763	Y705	4.59	pass
	NFKB	P19838	S536	114.61	pass

Figure S1. Luminex phospho-proteomics results
(A) Venn diagram showing the phosphorylated proteins detected in the protein lysates of the control static (pink), control dynamic (purple), TNF static (blue), and TNF dynamic (green) conditions. (B) Average Net MFI of protein lysates in each condition. Proteins are listed in alphabetical order, including AKT1, AKTS1, CHK2, CREB1, EGFR, FAK, GSK3, HSPB1, IKBA, JUN, MARCKS, MEK1, MTOR, NFκB, p38, p53, PTN11, RSK1, SMAD3, STAT3.
Shown are the medians. Data is not included in Results due to low donor number (n = 2).

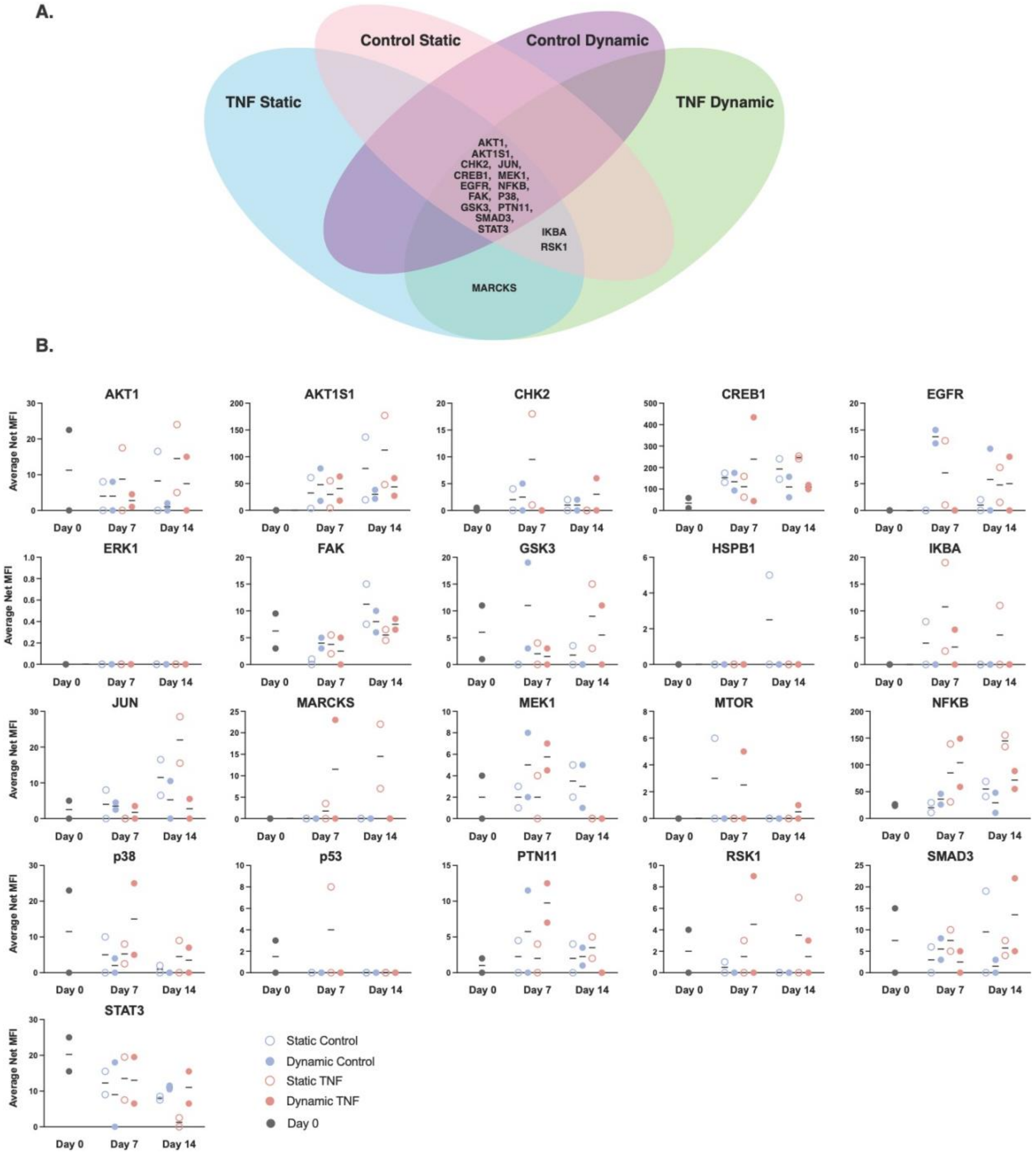

Supplement: Supplementary file 1 — Supplementary Material 1 [file 41598_2025_538_MOESM1_ESM.pdf]
